# Vectorial secretion of interleukin-8 mediates autocrine signalling in intestinal epithelial cells via apically located CXCR1

**DOI:** 10.1186/1756-0500-6-431

**Published:** 2013-10-28

**Authors:** Oriana Rossi, Jurgen Karczewski, Ellen H Stolte, Robert J M Brummer, Michiel A van Nieuwenhoven, Marjolein Meijerink, Joost R J van Neerven, Sven C D van Ijzendoorn, Peter van Baarlen, Jerry M Wells

**Affiliations:** 1 Host-Microbe Interactomics Group, ASG, University of Wageningen, Wageningen, The Netherlands; 2 Department of Gastroenterology, Örebro University, Örebro, Sweden; 3 FrieslandCampina, Amersfoort, The Netherlands; 4 Department of Cell Biology, Section Membrane Cell Biology, University Medical Center Groningen, Groningen, The Netherlands

**Keywords:** Intestinal epithelial cells, Interleukin-8, CXCR1, Caco-2, Epithelial repair, Autocrine

## Abstract

**Background:**

In the intestinal mucosa, several adaptations of TLR signalling have evolved to avoid chronic inflammatory responses to the presence of commensal microbes. Here we investigated whether polarized monolayers of intestinal epithelial cells might regulate inflammatory responses by secreting IL-8 in a vectorial fashion (i.e. apical versus basolateral) depending on the location of the TLR stimulus.

**Results:**

In the Caco-2 BBE model of polarized villus-like epithelium, apical stimulation with TLR2 and TLR5 ligands resulted in the apical secretion of IL-8. The CXCR1 receptor for IL-8 was expressed only on the apical membrane of Caco-2 BBE cells and differentiated epithelial cells in the human small intestine and colon. Transcriptome analyses revealed that Caco-2 BBE cells respond to stimulation with IL-8 supporting the hypothesis that IL-8 induces G protein-coupled receptor signalling.

**Conclusions:**

These results show that IL-8 induces autocrine signalling via an apical CXCR1 in Caco-2 BBE intestinal epithelial cells and that this receptor is also expressed on the apical surface of differentiated human intestinal epithelial cells *in vivo*, suggesting an autocrine function for IL-8 secreted in the lumen.

## Background

Intestinal epithelial cells (IECs) play a key role in the inflammatory response to colonizing or invading microorganisms via the secretion of interleukin-8 (IL-8). The chemokine IL-8 recruits neutrophils from the vasculature to sites of infection or tissue injury [1]. IL-8 can signal through two receptors, CXCR1 and CXCR2 both of which are members of the G-protein-coupled receptor (GPCR) family [2]. In neutrophils, IL-8 binding to CXCR1 triggers G protein-coupled signalling and formation of second messengers that mediate cellular migration, exocytosis of effectors and a respiratory burst to facilitate oxygen-dependent killing of phagocytosed microorganisms [2]. CXCR1 is expressed in the human IEC line Caco-2 BBE [3], which is in agreement with our own microarray data using Caco-2 BBE cell mRNA (see below). Additionally, IL-8 has been shown to induce Caco-2 BBE cell migration after wounding in a CXCR1-dependent manner [3]. The intestinal epithelium is structurally and functionally polarized, with tight junctions (TJs) preventing the diffusion of receptors, transporters and enzymes between the apical and basolateral membranes [4]. Several cell receptors are differentially expressed on the apical and basolateral membranes of polarized IECs including Toll-like receptors (TLRs), cytokine receptors and chemokine receptors [5,6]. In the human intestinal epithelium, TLR signalling is known to induce secretion of IL-8 in a nuclear factor (NF)-κB-dependent manner and TLR2, TLR5 and TLR9 are expressed on the apical poles of IECs depending on their location and differentiation status [5-8]. Interestingly, IL-8 has been measured in the colon milieu of healthy volunteers using a dialysis bag inserted in the rectum [9] suggesting that it might be apically secreted by colonic epithelial cells. Furthermore, IL-8 is present in significant amounts in breast milk [10]. However, it is not known if CXCR1 is expressed on the apical or basolateral poles in polarized Caco-2 BBE cells or human intestinal tissue or whether the direction of TLR signalling influences the direction of IL-8 secretion.

Here we investigated IL-8 secretion in response to TLR signalling using intestinal Caco-2 BBE cells grown in the Transwell system. In this established model, Caco-2 BBE monolayers differentiate into enterocytes [11] possessing intact TJs and distinct apical and basolateral membranes. We investigated whether the location (i.e. apical or basolateral) of TLR agonists influenced the production and direction of IL-8 secretion. Additionally, we determined the localization of the CXCR1 receptor in Caco-2 BBE cells and human intestinal tissue samples and investigated the role of IL-8 autocrine signalling using transcriptomics.

## Results

### Secretion of IL-8 in polarised Caco-2 BBE cell monolayers

**Figure 1:**
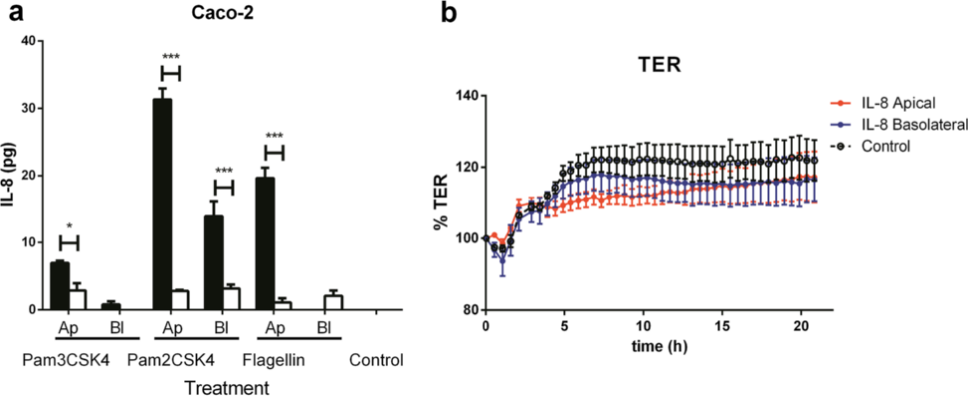
**Secretion of IL-8 in polarized Caco-2 BBE intestinal epithelial cells in response to Toll-like receptor ligands.** Caco-2 BBE **(a)** monolayers were stimulated for 24 hours from the apical (Ap treatment) or the basolateral compartment (Bl treatment) with: Pam3CSK4 (20 μg/ml), Pam2CSK4 (10 μg/ml) and flagellin (2 μg/ml). Amounts of IL-8 secreted in the apical (black bars) and basolateral (white bars) compartments are normalized to the volume of supernatant in each chamber. Error bars represent SEM (n=3). **(b)** IL-8 does not alter trans-epithelial electrical resistance (TER) in Caco-2 BBE monolayers. TER of Caco-2 BBE polarized monolayers untreated (black) or incubated with 100 pg/ml IL-8 on the apical (blue) or the basolateral side (red) for 20 hours. This concentration of IL-8 was chosen because it elicited a differential transcriptional response in Caco-2 BBE cells. TER values were normalized to the initial TER value (100%) and the differences between apical and basolateral treatment and control were not statistically significant. Error bars represent SEM (n=4); *p<0.05, **p<0.01, ***p<0.001.

Stimulation of polarized Caco-2 BBE cells with Pam2CSK4 and Pam3CSK4, agonists for TLR2/6 and TLR2/1 from the apical or basolateral sides induced secretion of IL-8 predominantly into the apical compartment regardless of the location of the stimulus, although basolateral stimulation induced smaller amounts of IL-8 compared to apical stimulation. In response to apical stimulation with the TLR5 agonist flagellin, IL-8 was secreted into the apical compartment while basolateral stimulation induced very small amounts of IL-8 only in the basolateral compartment (Figure 1a).

We ruled out the possibility that IL-8 could diffuse across the cell monolayers by adding it either to the apical or basolateral compartment and determining the concentration in each compartment after 24 hours. In the absence of cell monolayer, IL-8 rapidly equilibrated to an equal concentration in both the apical and basolateral chambers (not shown). Additionally, we demonstrated that the TJs remained intact throughout the experiment (not shown) and after apical or basolateral addition of IL-8 to Caco-2 BBE monolayers by measuring the trans-epithelial electrical resistance (TER, Figure 1b).

### CXCR1 is expressed on the apical membrane of polarized Caco-2 BBE cells

Given our observations on the apical secretion of IL-8 in IEC lines, we hypothesized that the localization of CXCR1 in polarized epithelium might be an important factor regulating IL-8-mediated autocrine signalling.

**Figure 2:**
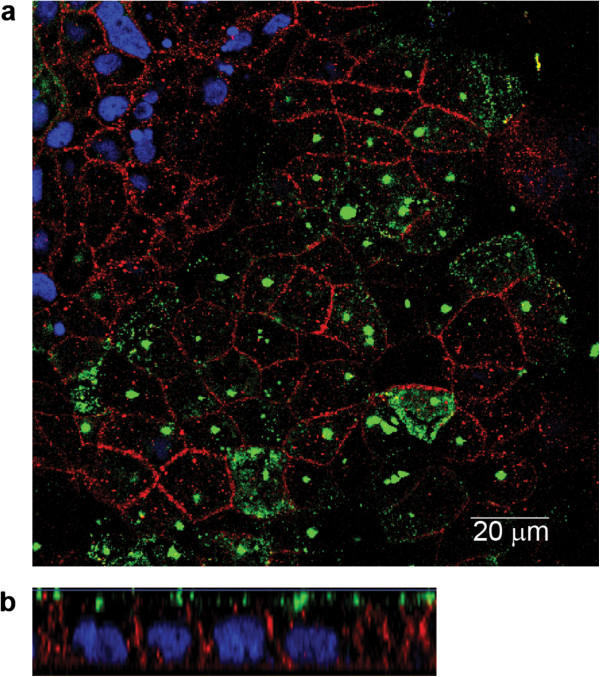
**Caco-2 BBE monolayers express CXCR1 on the apical surface.** The cellular localization of CXCR1 in Caco-2 BBE polarized monolayers visualized by immunofluorescent detection and confocal microscopy; anti-occludin (red), DRAQ5 (blue) and anti-CXCR1 (green). CXCR1 was expressed on the apical side of Caco-2 BBE monolayers. (x-y **(a)** and x-z **(b)** sections).

CXCR1 was localized to the apical pole of polarized Caco-2 BBE cells by confocal microscopy (Figure 2a and b). The speckled or ‘patchy’ staining for CXCR1 is suggestive of its localization in (macro)-domains, possibly microvilli. Other GPCRs and their effector proteins have shown patchy staining in cytoplasmic membrane domains of epithelial cells [12,13]. No CXCR1 staining was observed using an isotope control of the primary antibody at the same concentration as the anti-CXCR1 antibody or the secondary antibody alone as a control, indicating that fluorescence was not due to non-specific antibody binding (not shown).

### In human duodenal and colonic tissues, CXCR1 is expressed on the apical surface of differentiated epithelial cells and is absent in the crypts

To investigate the localization of CXCR1 in the human intestine, tissue samples of human colon and duodenum were collected from healthy volunteers, sectioned at low temperature, processed and stained.

**Figure 3:**
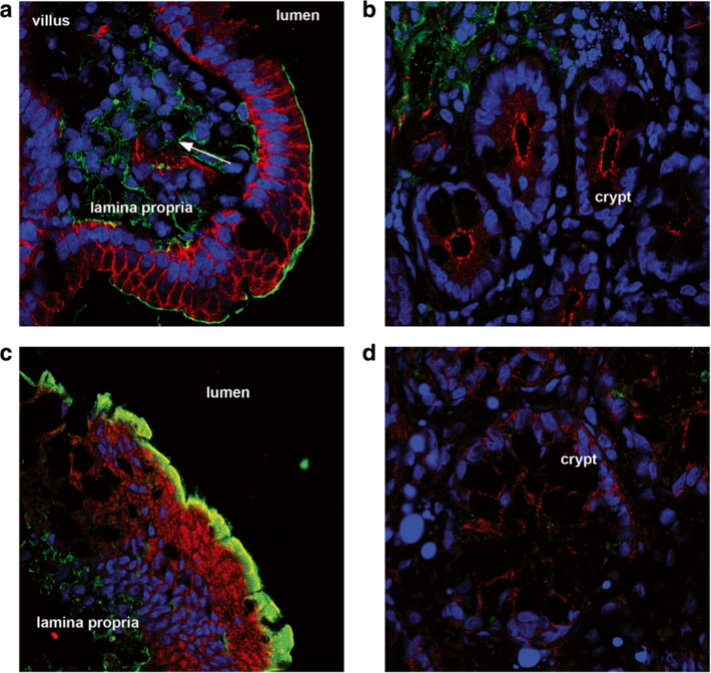
**In human duodenal and colonic tissues, CXCR1 is expressed on the apical surface of villus enterocytes and is absent in the crypts.** Duodenum and colon biopsies stained with anti-occludin (red), DRAQ5 (blue) and anti-CXCR1 (green). In villus enterocytes, CXCR1 is located on the apical membrane (sections of duodenum **(a)** and colon **(c)**), and surrounding the nuclei of cells in the lamina propria (**a**, arrow). CXCR1 is absent in the enterocytes lining the crypts of duodenum **(b)** and colon **(d)**.

The absorptive enterocytes forming the epithelium and the enterocytes lining the crypts, were revealed by staining for the TJ protein occludin (red) and nuclear DNA (blue, Figure 3a-d). In the duodenum, CXCR1 was detected on the apical membrane of villus epithelial cells and not in crypt epithelium (green, Figure 3a and b). Similarly, in human colonic tissue CXCR1 was detected on the top of the epithelium and not in crypt enterocytes (green, Figure 3c and d). The surface of immune cells present in the lamina propria in colonic and duodenal tissues also stained positive for CXCR1 (Figure 3a and c).

These results are in agreement with the known surface expression of CXCR1 in immune cells such as macrophages, dendritic cells, T cells and natural killer cells and our results with Caco-2 BBE cells.

### IL-8 autocrine signalling results in the differential expression of 859 genes

The autocrine role of IL-8 on IECs was investigated by measuring genome-wide transcriptome responses of Caco-2 BBE cells to IL-8 using the Affymetrix human whole genome expression microarrays. Polarized Caco-2 BBE cell monolayers were stimulated on the apical surface with IL-8 and, after 6 hours, total RNA was isolated for hybridization to whole genome expression microarrays. Quality control of the hybridisations and primary data analysis were performed according to strict criteria (Additional file 1) to ensure that the array data were of the highest possible quality.

Stimulation of Caco-2 BBE cells with IL-8 resulted in the differential expression of 859 genes (P<0.02; Additional file 2: Table S1). As gene set comparisons and pathway reconstruction from differentially expressed genes are far more informative than their tabulated up- or down-regulation [14], four complementary *in silico* approaches were employed to deduce the biological significance of the array data set. These approaches relate changes in gene expression to cellular pathways and processes modulated by transcriptional networks, and show how these are interacting towards common cellular processes.

### Gene ontology enrichment analysis and protein-protein interaction network of differentially expressed genes

**Figure 4:**
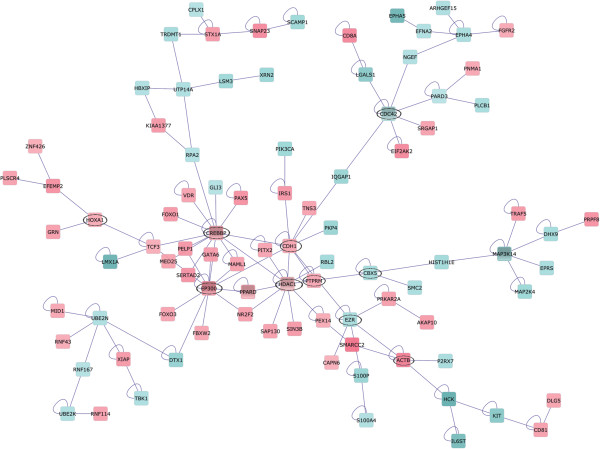
**Network showing interactions between proteins encoded by genes differentially expressed after stimulation of Caco-2 BBE cell monolayers with IL-8.** This network includes only differentially expressed genes that encode for proteins interacting with other proteins. Up-regulated genes are shown in shades of red and down-regulated genes in shades of blue; the intensity reflecting the fold-change. Note that the genes encoding the most connected proteins in the central part of the network (CREBBP, EP300, PPARD and HDAC1) were all up-regulated; these genes encode transcriptional regulators involved in lipid metabolism and cell differentiation. The network illustrates the core transcriptome changes modulating downstream processes including cell migration, signalling and differentiation.

To identify the strongest transcriptome changes of Caco-2 BBE cells in response to IL-8, we first performed Gene Ontology (GO) enrichment analysis using the software tool ErmineJ. This software identifies functional groups in which the differentially expressed genes are statistically overrepresented. Caco-2 BBE genes involved in lipoprotein biosynthesis, protein transport and secretion, response to virus, regulation of transcription and the cell cycle showed the strongest changes upon stimulation by IL-8 (Additional file 3: Table S2). To identify the gene regulatory networks driving these changes, a protein-protein interaction network was generated using the software platform Cytoscape [15] and overlaid with expression data using the differentially expressed genes as input. In this protein-protein interaction network, the nodes represent proteins encoded by genes that were differentially expressed. Inspection of this network (Figure 4) showed that the genes with the higher number of interactions were as follows: cAMP responsive element binding (CREB) binding protein (CREBBP), E1A-binding protein p300 (EP300), histone deacetylase 1 (HDAC1), chromobox homologue 5 (CBX5), epithelial E-cadherin (CDH1), ezrin (EZR), beta-actin (ACTB) and cell division cycle 42 (CDC42).

The genes CREBBP, EP300, CBX5, and HDAC1 encode coactivators and chromatin modification enzymes required for remodelling of tissue-specific gene loci and the activities of key transcriptional regulators during cell growth and differentiation [16,17]. CREBBP was up-regulated (red, Figure 4) in response to IL-8 and its overexpression in primary cultures of smooth muscle cells inhibits cell cycle progression [18]. The genes CDC42 and EZR were down-regulated in IL-8 treated cells suggesting decreased proliferation [19,20]. The genes CDH1, PTPRM (protein tyrosine phosphatase, receptor type, M), EZR and ACTB encode structural proteins that mediate cell-cell contact (CDH1 and PTPRM), define cell shape (ACTB) or act as intermediates between plasma membrane and actin cytoskeleton (EZR). Decreased EZR expression (discussed above) was previously linked to up-regulation of E-cadherin (CDH1) as observed in this study. HOXA1, a downstream effector of E-cadherin-directed signalling, was up-regulated by IL-8 (red, Figure 4), presumably due increased E-cadherin ligation. Taken together, the protein network responses suggest that IL-8 signalling is involved in the regulation of cell differentiation (structural proteins and cell-cell contact signalling) and lipid metabolism but not proliferation.

### Gene ontology categories network of differentially transcribed genes

**Figure 5:**
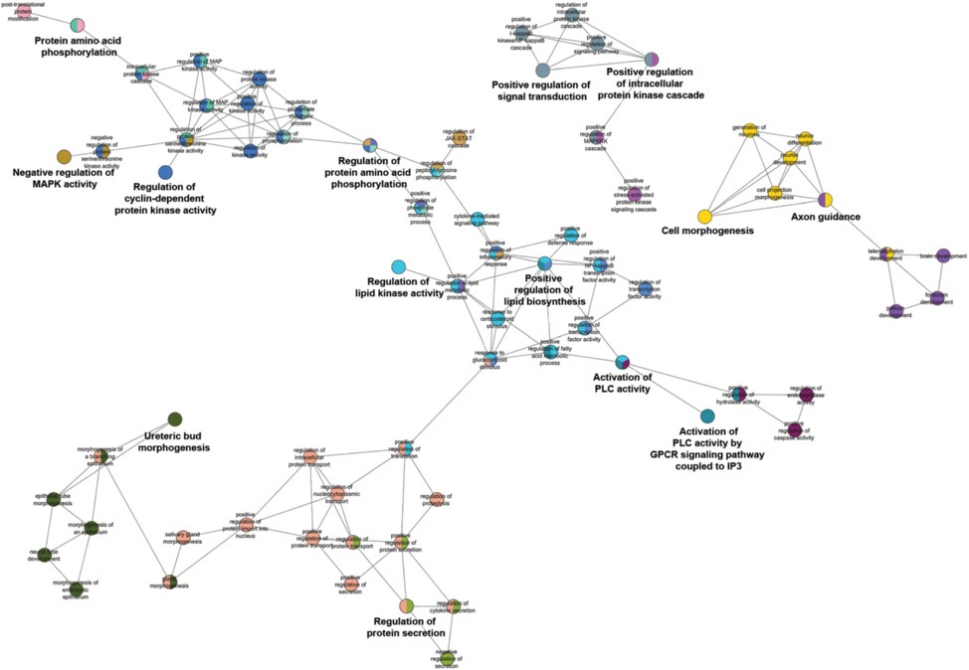
**Network showing linked GO categories representing the functional annotation of the genes that were differentially transcribed in Caco-2 BBE cells after stimulation with IL-8.** Gene ontology (GO) terms make up a formal vocabulary to describe gene functions. This network illustrates the biological processes corresponding to the transcriptome changes. Note that these GO categories exemplify cell migration, signalling and differentiation and therefore correlate well with the core protein-protein interaction network depicted in Figure 4 (see text for details). MAPK, MAP kinase; PLC, phospholipase C; GPCR, G protein-coupled receptor; IP3, inositol 1,4,5-trisphosphate.

To characterize further the processes that are regulated via these protein-protein interaction networks, we performed a GO enrichment analysis of all differentially regulated genes and visualized the interconnections between GO categories (Figure 5). The GO enrichment analysis identified three interconnected networks. The two smallest networks included GO categories involved in positive regulation of signal transduction and intracellular protein kinase cascade (top centre in Figure 5) and GO categories involved in cell morphogenesis and development (centre right in Figure 5). The largest network consisted of a central region that included GO categories involved in lipid biosynthetic processes including lipid kinase activity. This central region was connected to sub-networks consisting of GO categories involved in cyclin-dependent protein kinase activity and MAP kinase activity (top left in Figure 5), protein secretion and epithelial morphogenesis (bottom left/centre in Figure 5) and activation of phospholipase C (PLC) activity (bottom centre/right in Figure 5) which results from GPCR signalling. The latter was expected, as it is known that CXCR1 is a GPCR. These GO categories correlated well with the enriched categories found using ErmineJ.

## Discussion

In IECs, IL-8 expression is known to be induced by proinflammatory cytokines, TLR signalling and cellular stress [2,21]. Basolateral secretion of IL-8 plays a role in the recruitment of neutrophils from the vasculature to sites of infection or tissue injury [1]. IL-8 can be measured in rectal dialysates of healthy subjects [9] suggesting that it can be apically secreted in the intestine. The polarized secretion of IL-8, IL-1 and IL-6 has been reported in epithelial cell lines of different tissue origin [22-24]. However, it is not known if TLR signalling can induce vectorial secretion of IL-8 in IECs. To investigate whether IECs secrete IL-8 apically in response to TLR signalling, we stimulated polarised Caco-2 BBE cell monolayers grown in the Transwell system with TLR agonists applied from the apical or basolateral side and we measured the concentration of IL-8 in the two compartments. Caco-2 BBE cells were most responsive to TLR2/1, TLR2/6 and TLR5 agonists applied to the apical membrane (Figure 1a) which is compatible with the apical localization of TLR2 and TLR5 in humans and Caco-2 cells [7,8]. Additionally, in Caco-2 BBE cells, most of the IL-8 secreted in response to TLR agonists was located in the apical compartment (Figure 1a).

These results show that Caco-2 BBE cells can secrete IL-8 apically in response to TLR2 and TLR5 signalling. We speculate that *in vivo* the steady-state activation of TLRs by microbe associated molecular patterns of commensal bacteria may account for the luminal IL-8 measured in healthy subjects [9]. Further support for this hypothesis comes from our data showing apical staining of the IL-8 receptor CXCR1 in Caco-2 BBE cells and human intestinal tissue (Figures 2 and 3). Previously, CXCR1 and CXCR2 were reported to be minimally, if at all, expressed in Caco-2 cells using RT-PCR [25]. However, in agreement with our own results, Sturm et al., demonstrated the expression of CXCR1 in Caco-2 cells [3]. The reasons for this discrepancy are not clear but may relate to the primer efficiency or the use of a different Caco-2 cell clone. It may also be due to the differentiation state of the cells as we only observed CXCR1 staining on differentiated epithelial cells and not in crypt epithelial cells in colon and ileal biospies *ex vivo.* In the intestine, apically secreted IL-8 might therefore have an autocrine function.

To gain more insights into the role of IL-8 autocrine signalling, we performed a microarray analysis of Caco-2 BBE cells treated with 100 pg/ml IL-8. Although some *in vitro* studies use up to 50 ng/ml of IL-8 for *in vitro* studies [3], we chose to use 100 pg/ml as this is similar to the amounts secreted by Caco-2 BBE monolayers in cell culture. It is difficult to predict the amount IL-8 present in the lumen of the healthy human intestine but rectal dialysates of healthy human subjects have been shown to contain approximately 20 pg/ml of IL-8 after 4 hours dialysis [9]. Statistical analysis of the transcriptome data revealed that 859 genes were differentially expressed. The differentially expressed genes were analysed using GO enrichment analysis to identify the functional categories of genes that were significantly altered by IL-8 signalling. Additionally, a protein-protein interaction network and GO categories network of differentially expressed genes was used to visualize the interactions between different processes altered by IL-8 treatment. The microarray data support the hypothesis that IL-8 induces GPCR signalling in Caco-2 BBE cells. The GO categories network indicates activation of PLC, formation of the second messenger inositol 1,4,5-trisphosphate, activation of phosphatidylinositol 3-kinase and consequent generation of phosphatidylinositol 3-phosphate (bottom right of GO categories network, Figure 5). The microarray analyses suggested that IL-8 induces an increase in cell-cell contact, cell adhesion and cell survival. In particular the gene network involved in E-cadherin regulation may play a role in the formation of epithelial cell-cell junctions and differentiation of epithelial cells after migration [26]. The E-cadherin–catenin complex has been shown to be a crucial to the regulation of cell adhesion, polarity, differentiation, migration, proliferation, and survival of IECs [27].

The microarray data also suggests that IL-8 is involved in the regulation of cell differentiation rather than proliferation. In this respect, it is also relevant that CXCR1 was expressed only on the differentiated and non-proliferative epithelial cells in the small intestine and colon and not in crypt enterocytes (Figure 3). Thus, IL-8 secreted in the lumen, for example in response to commensal antigens, would not have an effect on the differentiating cells that migrate upwards from the bottom of the crypts. Previously, IL-8 was reported to induce proliferation, differentiation and CXCR1-dependent migration in Caco-2 BBE cells [3,10]. Although the effect on of IL-8 on epithelial cell proliferation is controversial [3] and was not confirmed by our own microarray data, overall the published data and our own work suggests a role for IL-8 in epithelial restitution. Our observation on the apical location of CXCR1 in human intestinal tissues and our microarray data lend support to the hypothesis that IL-8 has an autocrine function *in vivo*. We speculate that apical secretion of IL-8 would help to initiate pathway responses in restitution prior to any potential loss of epithelial integrity, e.g. because of bacterial invasion or toxin production. In this respect, it is relevant that addition of IL-8 to the apical or basolateral side of an intact epithelial barrier had no significant effect on TER over a period of at least 20 hours (Figure 1b). This suggests that IL-8 mediated autocrine signalling would not affect the viability, permeability or integrity of an intact epithelium.

Interestingly, the effects of IL-8 on proliferation, differentiation and migration have also been shown using fetal IECs [10]. As significant concentrations of IL-8 are found in milk and amniotic fluid, it was suggested that IL-8 might have an additional role in the developing intestine [10]. A similar autocrine role has been proposed for CCL20, which is also induced by inflammatory pathways in epithelial cells. CCL20 binds to the CCR6 receptor on the apical pole of differentiated epithelial cells [12] and induces epithelial cell migration *in vitro*[28].

## Conclusion

In summary, we show for the first time that the IL-8 receptor CXCR1 is expressed on the apical membrane of differentiated epithelial cells in the human small intestine and colon. Furthermore, our data demonstrate that IL-8 is secreted from the apical pole of IEC lines in response to TLR signalling and suggest that polarized secretion of IL-8 may occur in differentiated epithelial cells of the villi in response to apical stimulation with microbe-associated molecular patterns.

## Methods

### Human materials

Human duodenum biopsies derive from the study of Troost et al., 2008 [29], this study was approved by the University Hospital Maastricht Ethical Committee, Colon biopsies were obtained from healthy subjects at the University Hospital Örebro, according to the study protocol from Brummer et al.: “Characterising intestinal microbiota and immune response in IBD, microscopic colitis and IBS”. The University Hospital Örebro Ethical committee (Dnr 2010/261) approved this study. Studies were conducted in full accordance with the principles of the ‘Declaration of Helsinki’ (52nd WMA General Assembly, Edinburgh, Scotland, October 2000). All subjects gave their written informed consent prior to their inclusion into the study.

### Cell culture

The Caco-2 BBE cell line (CRL 2102; American Type Culture Center, Manassas, VA) was maintained at 37°C in a humidified 5% CO_2_/95% O_2_ atmosphere. Caco-2 BBE cells were grown in DMEM (Invitrogen, Paisley, UK) containing Glutamax and supplemented with 10% fetal bovine serum (PAA laboratories, Colbe, Germany), 100 U/ml penicillin and 100 μg/ml streptomycin (Sigma, St. Louis, MO). Cells (between passage 55 and 74) were seeded at a density of 2.6 × 10^5^ cells/cm^2^ and grown for 14 days until they differentiated into polarized monolayers. After 14 days, the TER reached 600 to 800 Ohms/cm^2^ (Volt/Ohm-meter, World Precision Instruments, Sarasota, FL).

### Electrical resistance measurements in monolayer cell cultures

To measure the effects of IL-8 on epithelial permeability, polarized Caco-2 BBE monolayers grown in Transwell filter inserts were treated with 100 pg/ml recombinant human IL-8 (rhIL-8, R&D Systems) added to the apical or basolateral compartment and the TER was measured using a Cellzscope (Nanoanalytics) which allows the continuous and automated measurement of 24 individual filter inserts under cell culture conditions.

### IL-8 secretion

DMEM containing TLR ligands was added to cell monolayers on day 14 either in the apical or in the basolateral compartment. The stimuli were used at the following concentrations: 20 μg/ml Pam3CSK4, 10 μg/ml Pam2CSK4, 2 μg/ml flagellin (InvivoGen, San Diego, CA). After 24 hours, 75 μl samples of apical or basolateral supernatant was taken and the concentration of IL-8 was determined using a cytometric bead array (BD, San Diego, CA) following the manufacturer instruction and flow cytometry (FACS CantoII, BD). Similar findings were obtained in triplicate experiments.

### Confocal microscopy

Caco-2 BBE cell monolayers grown in transparent Transwell inserts for 14 days were fixed in 4% (weight/vol) paraformaldehyde and permeabilized with PBS containing 0.2% (vol/vol) Triton X100, 1% (vol/vol) normal goat serum and 0.1% (weight/vol) sodium azide. Samples were then incubated with primary antibodies, 1:250 of anti-occludin polyclonal antibody (Zymed, Invitrogen) and 1:100 dilution of monoclonal anti-human IL-8RA (i.e. CXCR1, R&D Systems) were added overnight at 4°C followed by a 2 hours incubation with Cy3-and Alexa 488-labeled secondary antibodies at room temperature. Nuclei were stained for 20 minutes using a dilution 1:500 of DRAQ5 (Enzo Life Sciences BVBA, Zandhoven, Belgium). Confocal images were obtained using a Zeiss LSM 510 system consisting of a Zeiss Axioskop with Plan Neofluar ×63 NA 1.3 oil objectives. Human duodenum and colon tissue sections (20 μm thick) were collected at −18°C on superfrost object glasses (Menzel-Glazer, Braunschweig, Germany) then processed and stained as described for Caco-2 BBE cells.

### RNA isolation and quality control

Caco-2 BBE monolayers grown for 14 days were treated with none or 100 pg/ml rhIL-8. After 6 hours treatment, total RNA was extracted from Caco-2 BBE cells using Trizol reagent (Invitrogen), purified using the RNeasy mini kit (Qiagen, Venlo, The Netherlands) and treated with DNase set (Qiagen) following the manufacturer’s instructions. RNA integrity was assessed using an Agilent 2100 Bioanalyzer (Agilent Technologies, Amsterdam, The Netherlands) with 6000 Nano Chips according to the manufacturer’s instructions. Only RNA samples showing intact bands corresponding to the 18S and 28S ribosomal RNA subunits, no chromosomal peaks or RNA degradation products, and an RNA integrity number (RIN) above 8.0 was used for microarray hybridization.

### Affymetrix GeneChip oligoarray hybridization and scanning

Total RNA, extracted from Caco-2 BBE cells was amplified and labelled using the GeneChip 3’ IVT Express kit (cat. no. 901229) according to the manufacturer’s specifications. Three independent experimental replicates per condition were used for total RNA extraction. The correspondingly labelled RNA was hybridised to a GeneChip Hu133 Plus2 array. Detailed methods for the labelling and subsequent hybridisations to the arrays are described in the eukaryotic section of the GeneChip Expression Analysis Technical Manual, Revision 3, from Affymetrix (Affimetrix, Santa Clara, CA), and are available upon request.

### Statistical and functional analyses of microarray data

Transcriptome datasets underwent rigorous checks for quality control before the pathway and network analyses. These software packages are part of Bioconductor project (http://www.bioconductor.org) and integrated in the automated MADMAX pipeline (https://madmax.bioinformatics.nl) as described in previous publications [30]. Using the same statistical approaches we showed a good correlation between microarray and quantitative PCR (qPCR) data [14]. Thus, we did not perform qPCR analyses on these samples.

Four complementary methods were used for the functional analysis of microarray expression data: ErmineJ (GO annotation enrichment or overrepresentation; [31]), Gene Set Enrichment Analysis [32], Ingenuity Pathway Analysis (IPA; http://www.ingenuity.com) and gene regulatory network reconstruction and analysis via Cytoscape [15]. Using these software tools, we performed: (i) identification of statistically supported overrepresentation of functional GO annotation [32], (ii) mapping of expression data onto pathways to determine their up- or down-regulation in a statistical meaningful way (IPA); (iii) projection of transcript fold-change values of co-expressed genes onto interaction maps of the corresponding proteins, and (iv) reconstruction of biologically meaningful cellular pathways and their mode of modulation (IPA and Cytoscape). Our approach of combining data outputs from one bioinformatics package with another to strengthen the analysis has been described previously [30].

### Supporting data

The microarray data and the raw cel files were deposited at GEO, platform number GSE30364 and can be accessed via the following link:

http://www.ncbi.nlm.nih.gov/geo/query/acc.cgi?token=ddafvqiigumcupm&acc=GSE30364.

## Competing interests

The authors declare that they have no competing interests.

## Authors’ contributions

OR, EK and JK performed the experiments. RB and MN collected the human biopsies. MM was involved in data collection. PvB performed the microarray data analysis. JvN and SvI contributed to the design of the experiments. OR and JW designed the experiments and wrote the manuscript. All the authors read and approved the final manuscript.

## Supplementary Material

Additional file 1 **Supplementary Quality Control Report: Outcome of quality control analyses performed using Bioconductor (http://www.bioconductor.org).** Click here for file

Additional file 2: Table S1 List of all differentially expressed genes (P<0.02), statistics and annotations. Click here for file

Additional file 3: Table S2 Gene ontology enrichment results as executed by ErmineJ for the transcriptomes altered after incubation of Caco-2 BBE cells with CXCL8. Click here for file
