# Supplementary material for: Vectorial secretion of interleukin-8 mediates autocrine signalling in intestinal epithelial cells via apically located CXCR1
Source: BMC Res Notes. 2013 Oct 28;6:431. doi: 10.1186/1756-0500-6-431 (PMC4231470; doi:10.1186/1756-0500-6-431)

Quality control report (BEFORE normalization only).
Date: Fri Jan 22 14:50:16 2010 / BioConductor version: 2.4 (R-2.9.0) / Platform: Linux / simpleaffy version: 2.20.0-1.
Scaling: target intensity (tgt) is: 100.000000

gapdh3 probeset is: AFFX-HUMGAPDH/M33197_3_at
gapdhM probeset is: AFFX-HUMGAPDH/M33197_M_at
gapdh5 probeset is: AFFX-HUMGAPDH/M33197_5_at
actin3 probeset is: AFFX-HSAC07/X00351_3_at
actinM probeset is: AFFX-HSAC07/X00351_M_at
actin5 probeset is: AFFX-HSAC07/X00351_5_at

Loaded Files	scaling factors	% present	average backgr.	minimum backgr.	maximum backgr.	BioB signal	BioB P/A	BioC signal	BioC P/A	BioD signal	BioD P/A	CreX signal	CreX P/A
Caco2-1.CEL	0.6103	47.3544	54.7234	52.2253	56.5260	8.3638	P	9.9410	P	12.2111	P	13.4152	P
Caco2-2.CEL	0.6150	47.6653	53.6337	51.1771	55.1821	8.2272	P	9.9113	P	12.1652	P	13.3054	P
Caco2-3.CEL	0.6102	46.1289	54.1638	51.8753	57.3492	8.2311	P	9.8927	P	12.1266	P	13.3543	P
Caco2-IL8-1.CEL	0.5200	48.7956	53.3060	51.5514	55.2819	8.4017	P	9.9270	P	12.1741	P	13.2812	P
Caco2-IL8-2.CEL	0.5487	48.3036	61.8904	59.4238	63.2917	8.3461	P	9.8889	P	12.1324	P	13.3358	P
Caco2-IL8-3.CEL	0.6237	46.7051	55.0188	52.0831	56.8531	8.3688	P	10.0140	P	12.2660	P	13.4268	P

Loaded Files	gapdh3 signal	gapdh3 P/A	gapdhM signal	gapdhM P/A	gapdh5 signal	gapdh5 P/A	actin3 signal	actin3 P/A	actinM signal	actinM P/A	actin5 signal	actin5 P/A
Caco2-1.CEL	12.7646	P	12.6245	P	12.7547	P	12.3206	P	11.6051	P	10.8000	P
Caco2-2.CEL	12.7723	P	12.6646	P	12.7802	P	12.3700	P	11.6432	P	10.6505	P
Caco2-3.CEL	12.7702	P	12.6400	P	12.7123	P	12.2459	P	11.4625	P	10.4529	P
Caco2-IL8-1.CEL	12.6162	P	12.4308	P	12.5965	P	12.1860	P	11.5492	P	10.9707	P
Caco2-IL8-2.CEL	12.6491	P	12.4776	P	12.6260	P	12.3079	P	11.6684	P	10.8873	P
Caco2-IL8-3.CEL	12.7866	P	12.6706	P	12.7594	P	12.3469	P	11.6768	P	10.8479	P

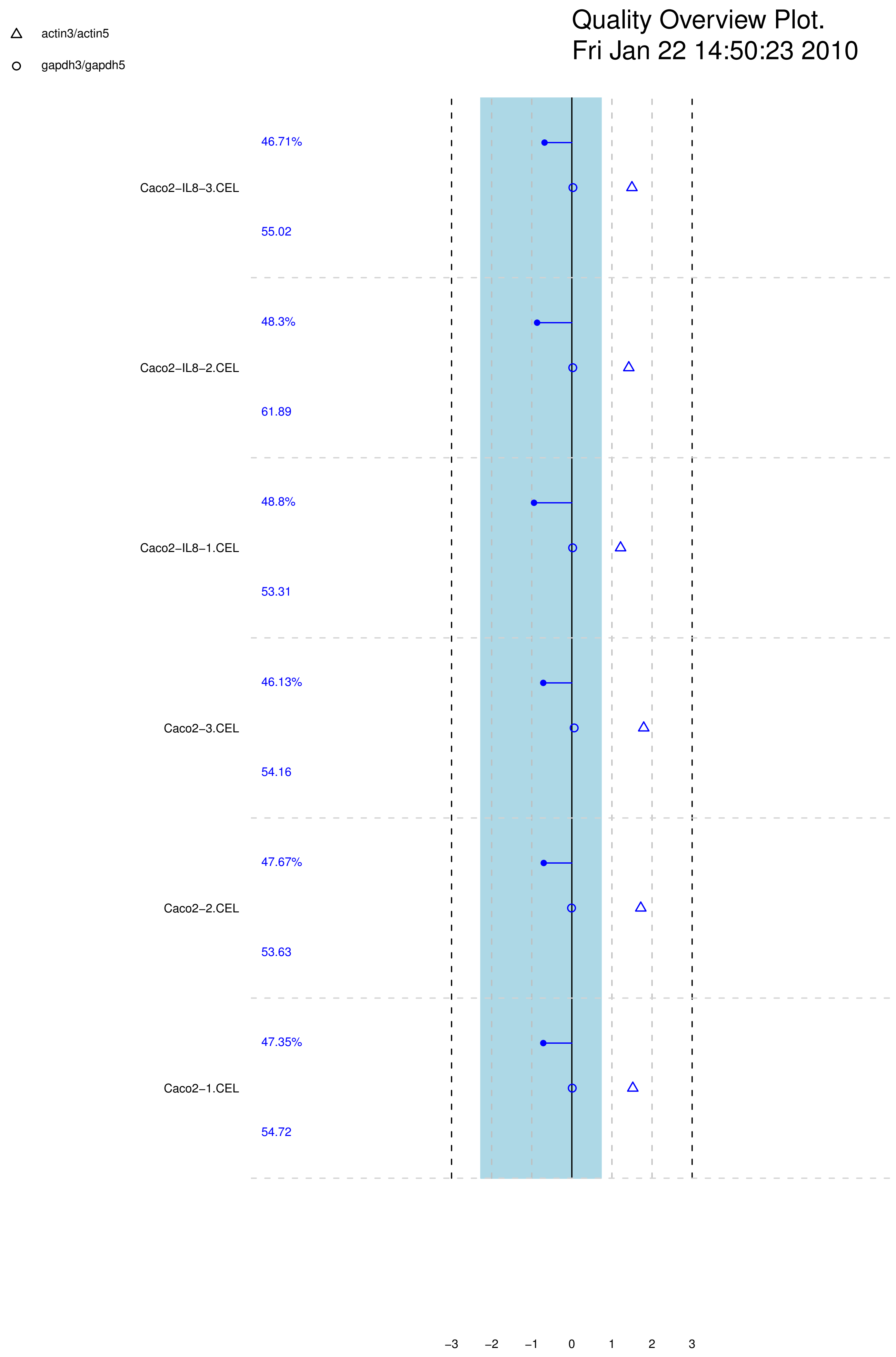

actin3/actin5
gapdh3/gapdh5
Quality Overview Plot.
Fri Jan 22 14:50:23 2010
46.71%
Caco2−IL8−3.CEL
55.02
48.3%
Caco2−IL8−2.CEL
61.89
48.8%
Caco2−IL8−1.CEL
53.31
46.13%
Caco2−3.CEL
54.16
47.67%
Caco2−2.CEL
53.63
47.35%
Caco2−1.CEL
54.72
−3 −2 −1 0 1 2 3

Caco2-1.CEL

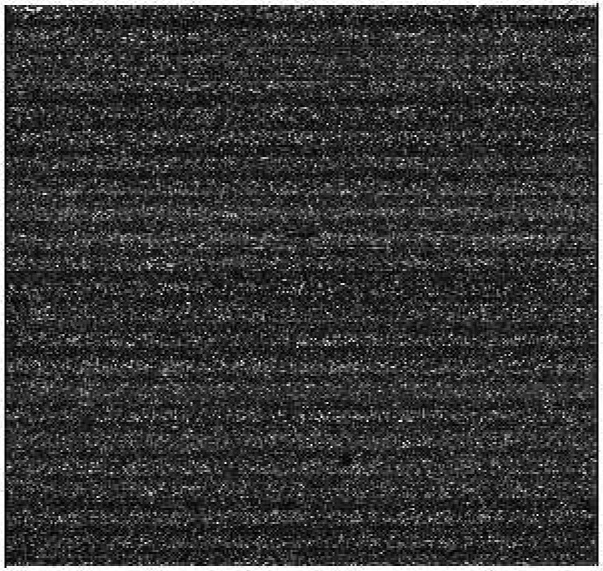

Raw image plot: 1/6 (Fri Jan 22 14:50:36 2010)

Caco2-2.CEL

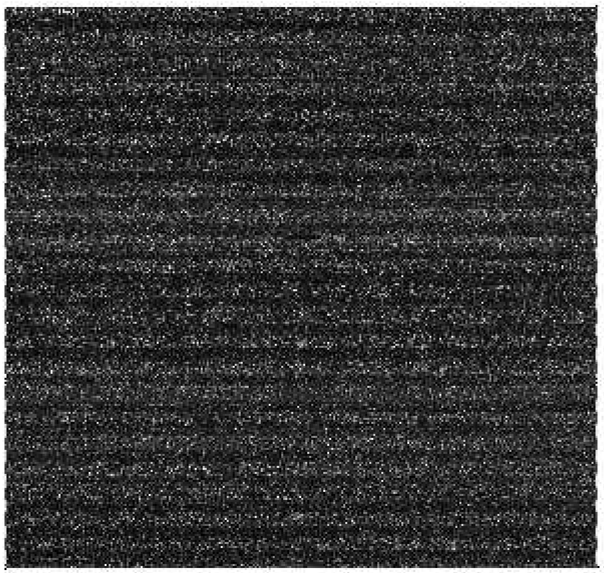

Raw image plot: 2/6 (Fri Jan 22 14:50:46 2010)

Caco2-3.CEL

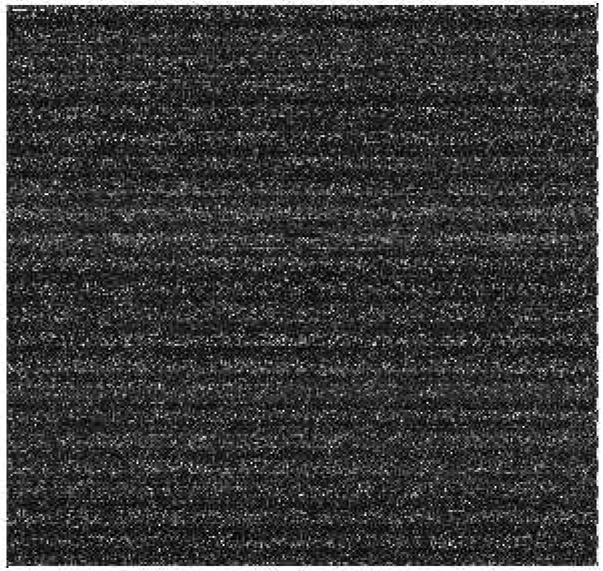

Raw image plot: 3/6 (Fri Jan 22 14:50:57 2010)

Caco2-IL8-1.CEL

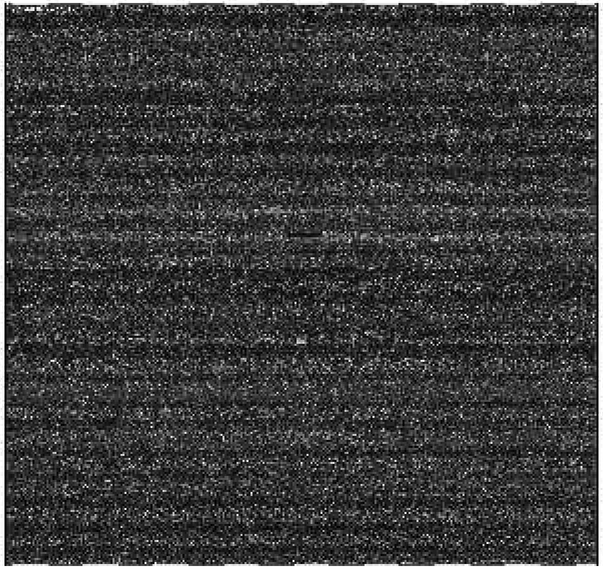

Raw image plot: 4/6 (Fri Jan 22 14:51:07 2010)

Caco2-IL8-2.CEL

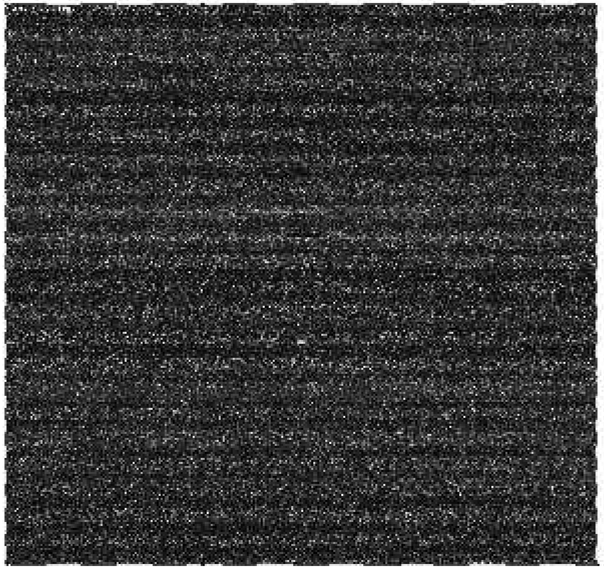

Raw image plot: 5/6 (Fri Jan 22 14:51:17 2010)

Caco2-IL8-3.CEL

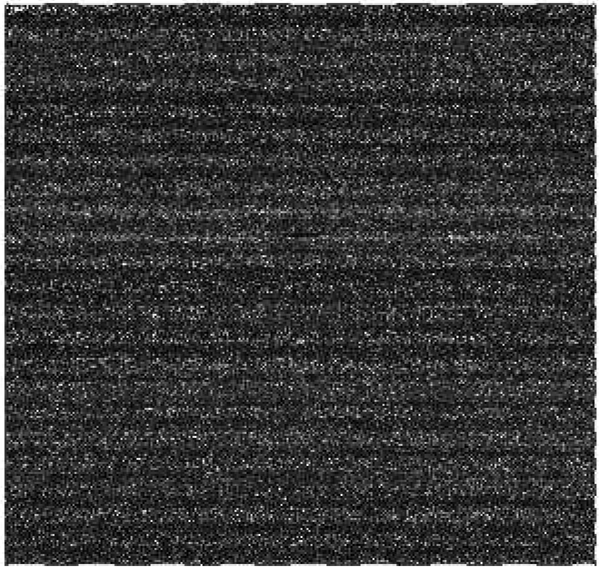

Raw image plot: 6/6 (Fri Jan 22 14:51:28 2010)

Caco2-1.CEL

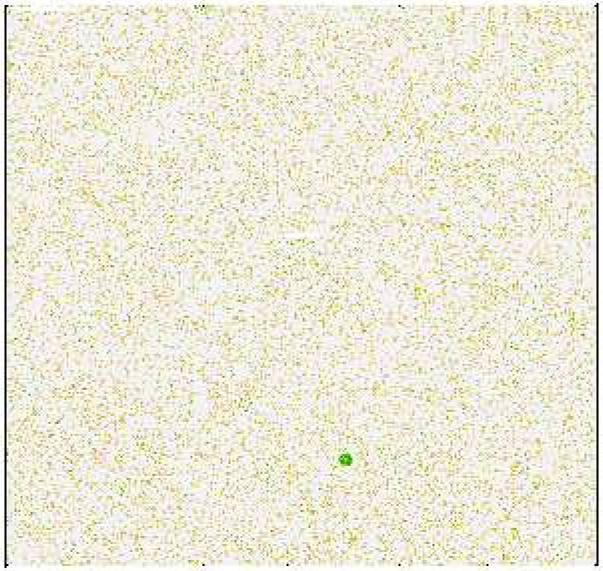

fitPLM weight image plot: 1/6 (Fri Jan 22 14:52:20 2010)

Caco2-2.CEL

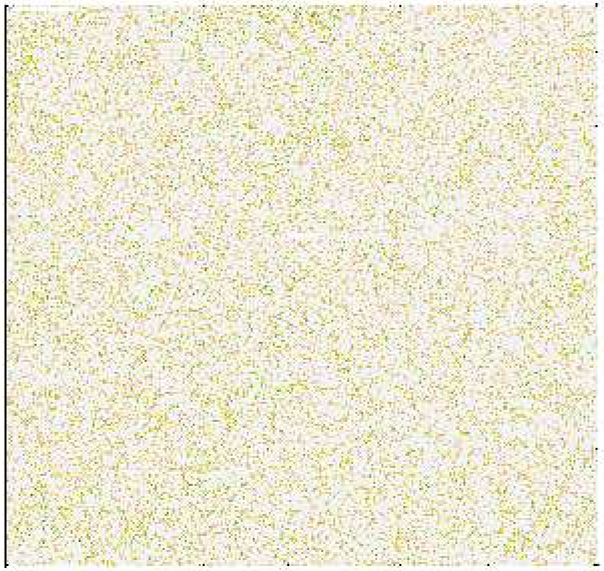

fitPLM weight image plot: 2/6 (Fri Jan 22 14:52:28 2010)

Caco2-3.CEL

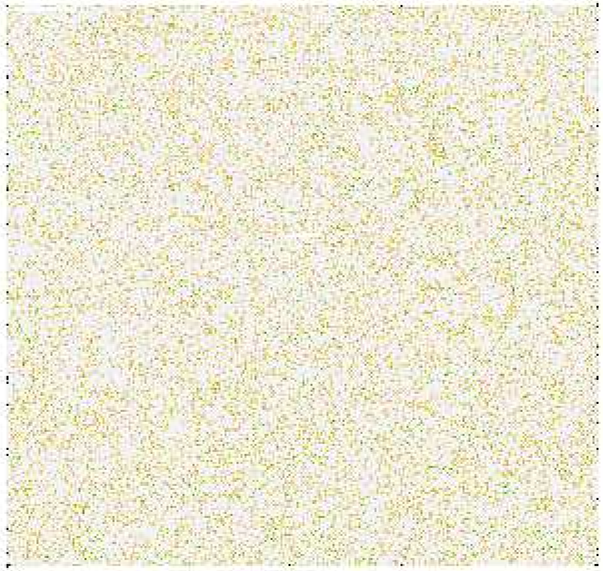

fitPLM weight image plot: 3/6 (Fri Jan 22 14:52:36 2010)

Caco2-IL8-1.CEL

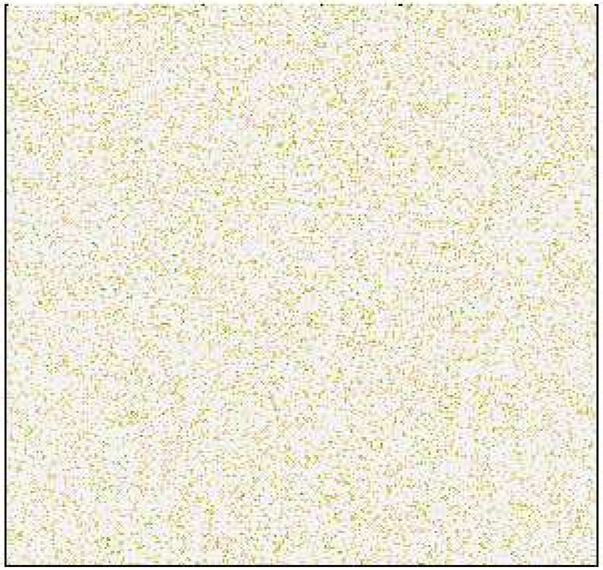

fitPLM weight image plot: 4/6 (Fri Jan 22 14:52:43 2010)

Caco2-IL8-2.CEL

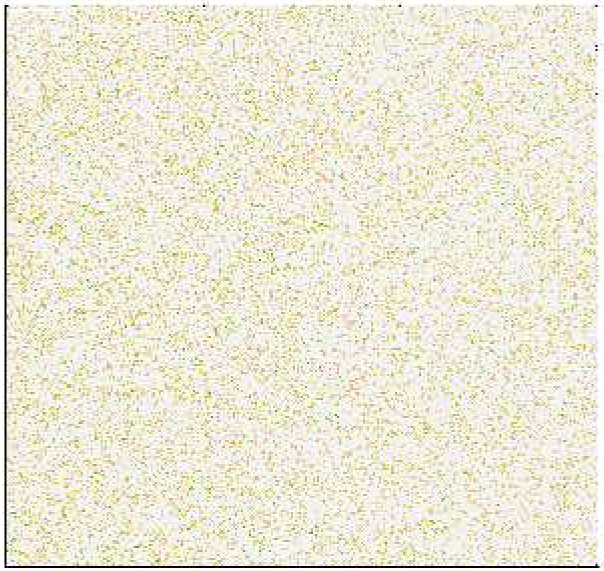

fitPLM weight image plot: 5/6 (Fri Jan 22 14:52:50 2010)

Caco2-IL8-3.CEL

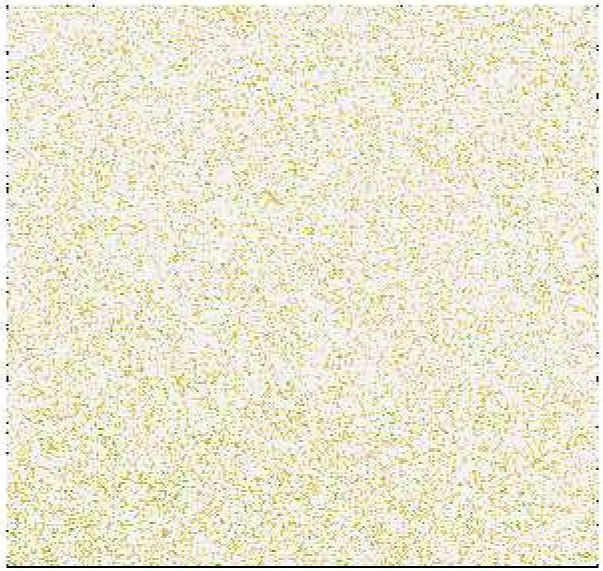

fitPLM weight image plot: 6/6 (Fri Jan 22 14:52:58 2010)

Caco2-1.CEL

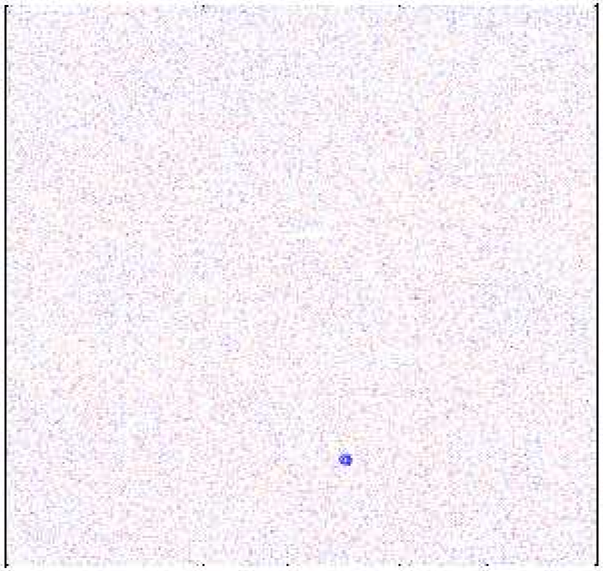

fitPLM residuals image plot: 1/6 (Fri Jan 22 14:53:08 2010)

Caco2-2.CEL

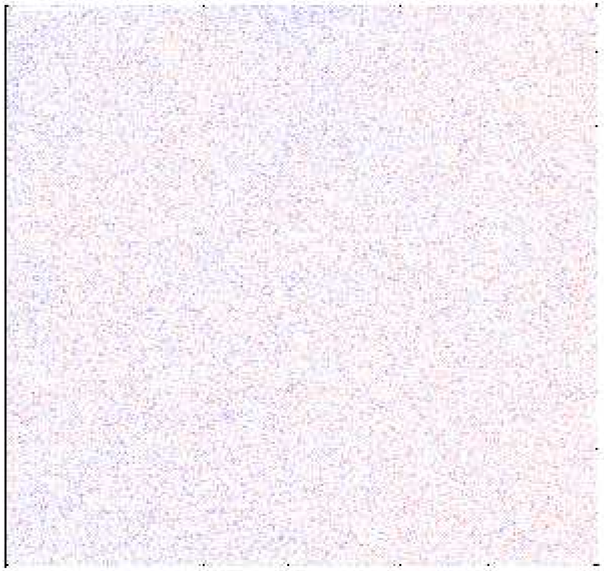

fitPLM residuals image plot: 2/6 (Fri Jan 22 14:53:17 2010)

Caco2-3.CEL

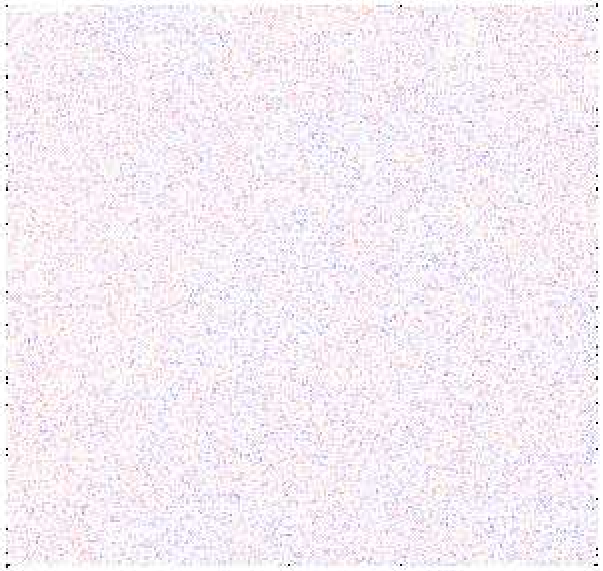

fitPLM residuals image plot: 3/6 (Fri Jan 22 14:53:25 2010)

Caco2-IL8-1.CEL

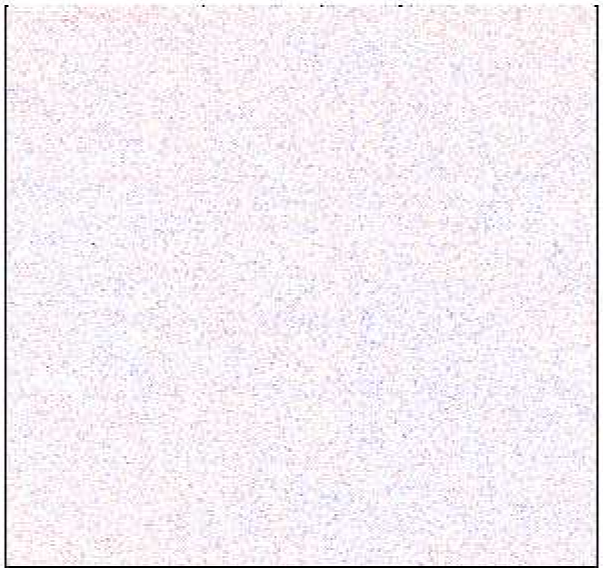

fitPLM residuals image plot: 4/6 (Fri Jan 22 14:53:34 2010)

Caco2-IL8-2.CEL

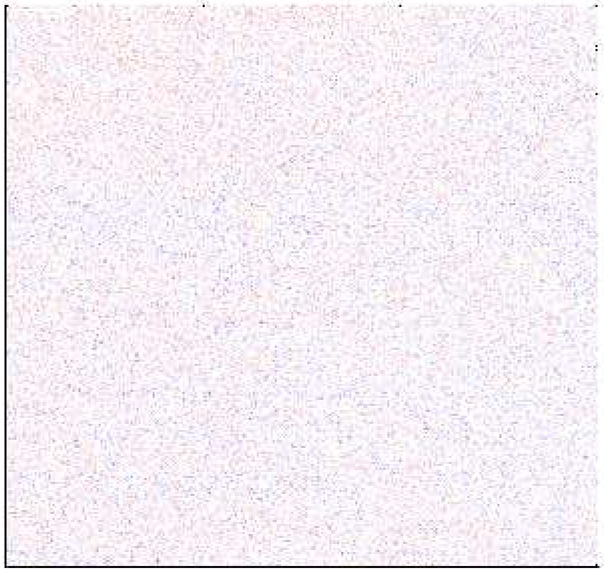

fitPLM residuals image plot: 5/6 (Fri Jan 22 14:53:41 2010)

Caco2-IL8-3.CEL

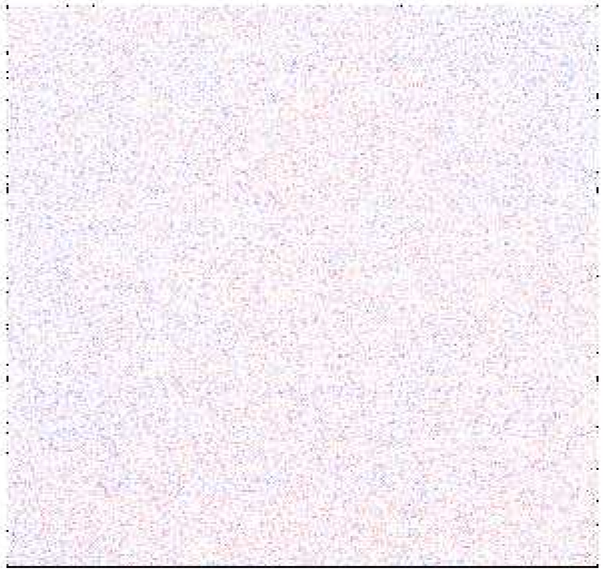

fitPLM residuals image plot: 6/6 (Fri Jan 22 14:53:49 2010)

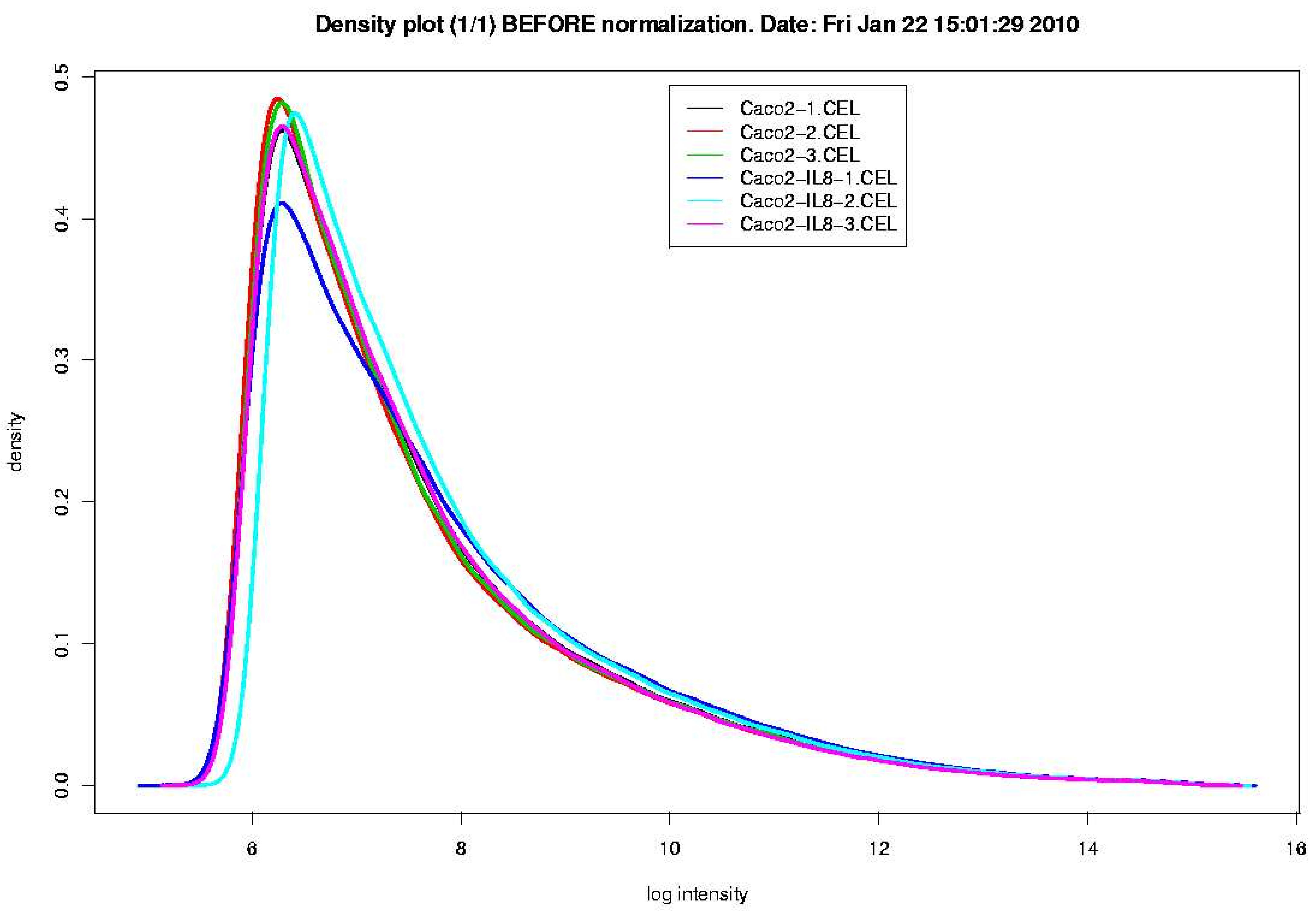

Density plot (1/1) BEFORE normalization. Date: Fri Jan 22 15:01:29 2010
Caco2−1.CEL
Caco2−2.CEL
Caco2−3.CEL
Caco2−IL8−1.CEL
Caco2−IL8−2.CEL
Caco2−IL8−3.CEL
density
0.0
0.1
0.2
0.3
0.4
0.5
log intensity
6
8
10
12
14
16

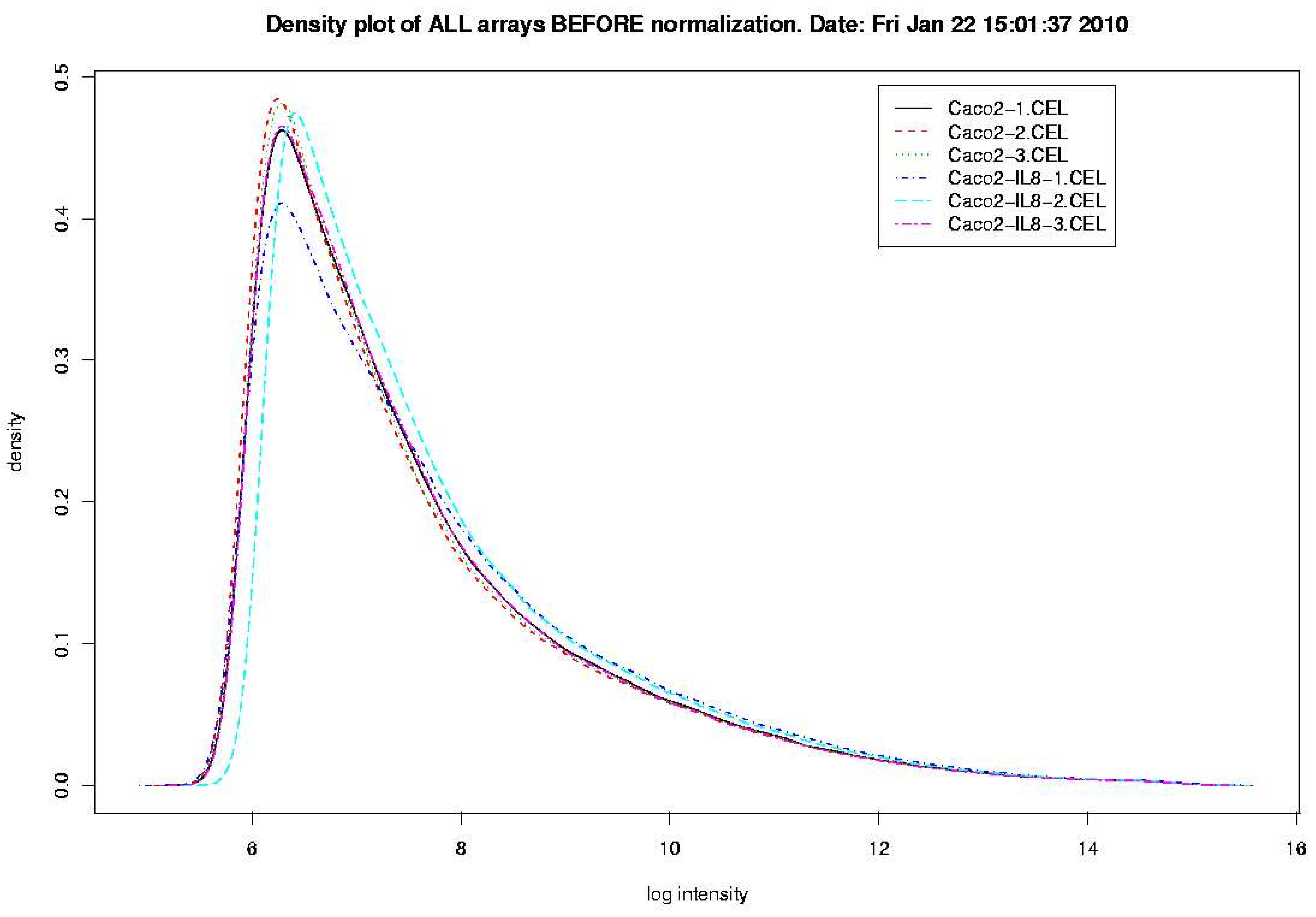

Density plot of ALL arrays BEFORE normalization. Date: Fri Jan 22 15:01:37 2010
density
0.0
0.1
0.2
0.3
0.4
0.5
log intensity
6
8
10
12
14
16
Caco2−1.CEL
Caco2−2.CEL
Caco2−3.CEL
Caco2−IL8−1.CEL
Caco2−IL8−2.CEL
Caco2−IL8−3.CEL

Boxplot BEFORE normalization. Date: Fri Jan 22 15:01:47 2010

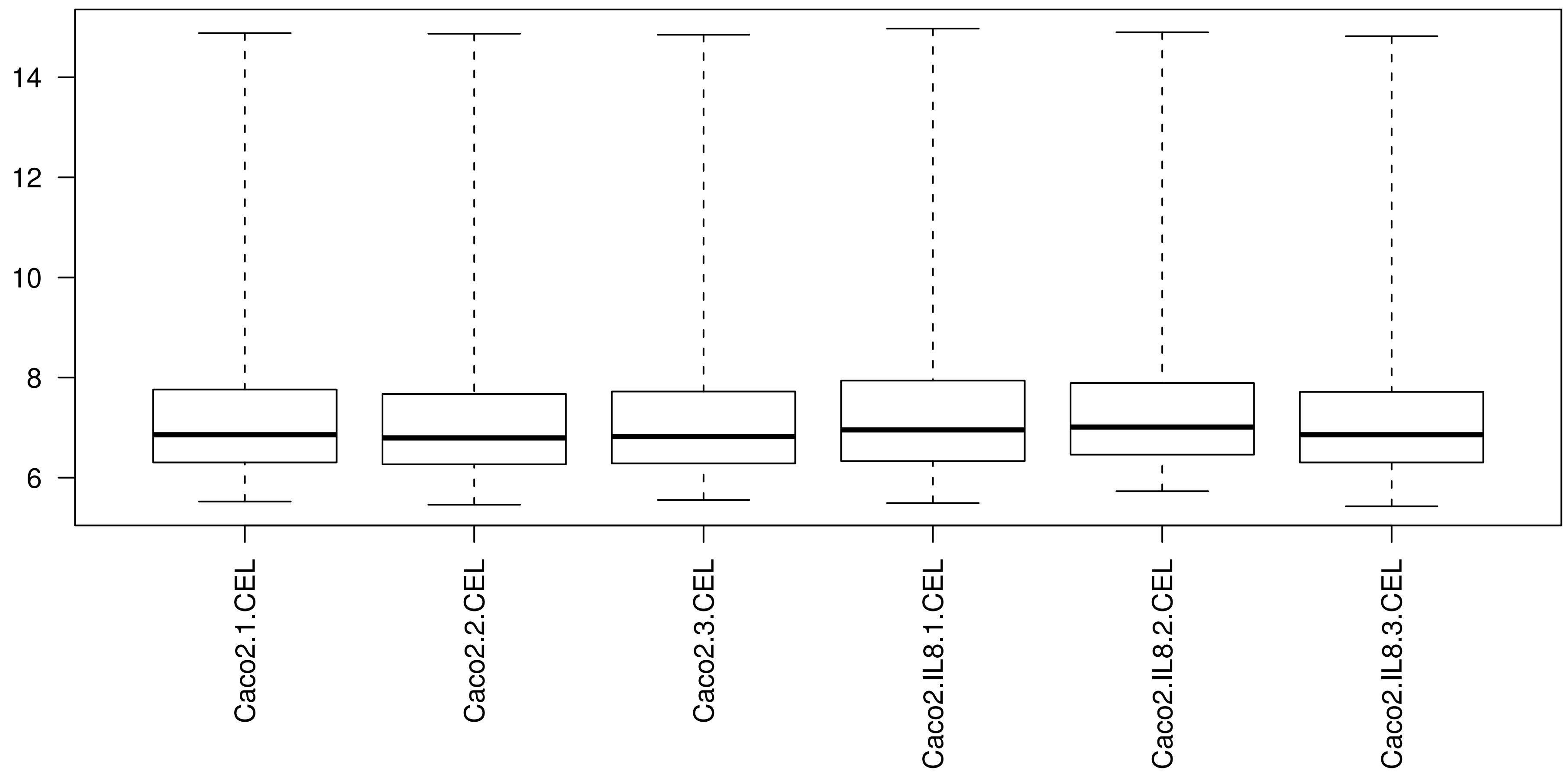

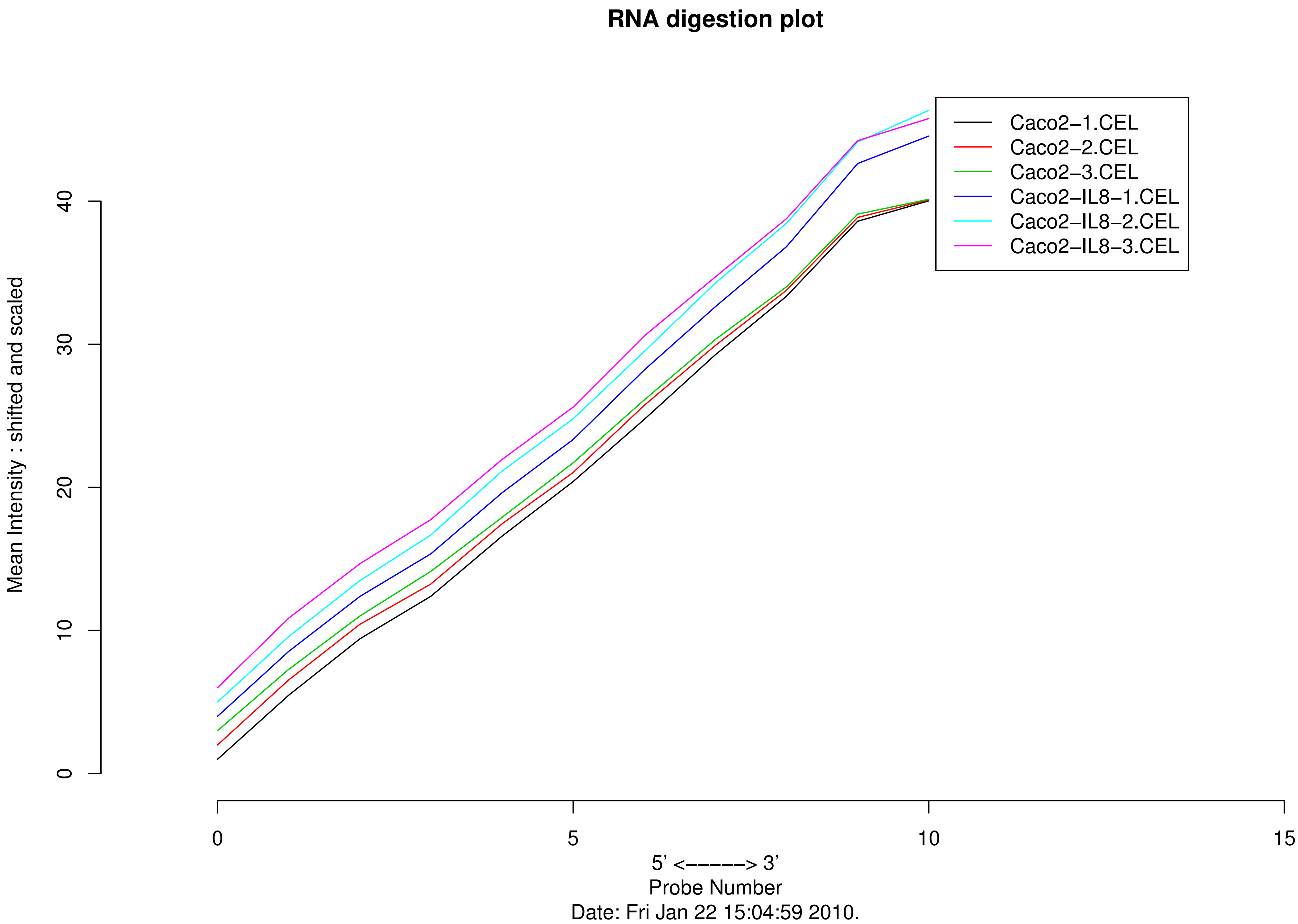
RNA digestion plot
Caco2−1.CEL
Caco2−2.CEL
Caco2−3.CEL
Caco2−IL8−1.CEL
Caco2−IL8−2.CEL
Caco2−IL8−3.CEL
Mean Intensity : shifted and scaled
0
10
20
30
40
0
5
10
15
5’ <−−−−−> 3’
Probe Number
Date: Fri Jan 22 15:04:59 2010.

Array−Array Intensity Correlation: Correlation plot BEFORE normalisation (Fri Jan 22 15:06:23 2010).

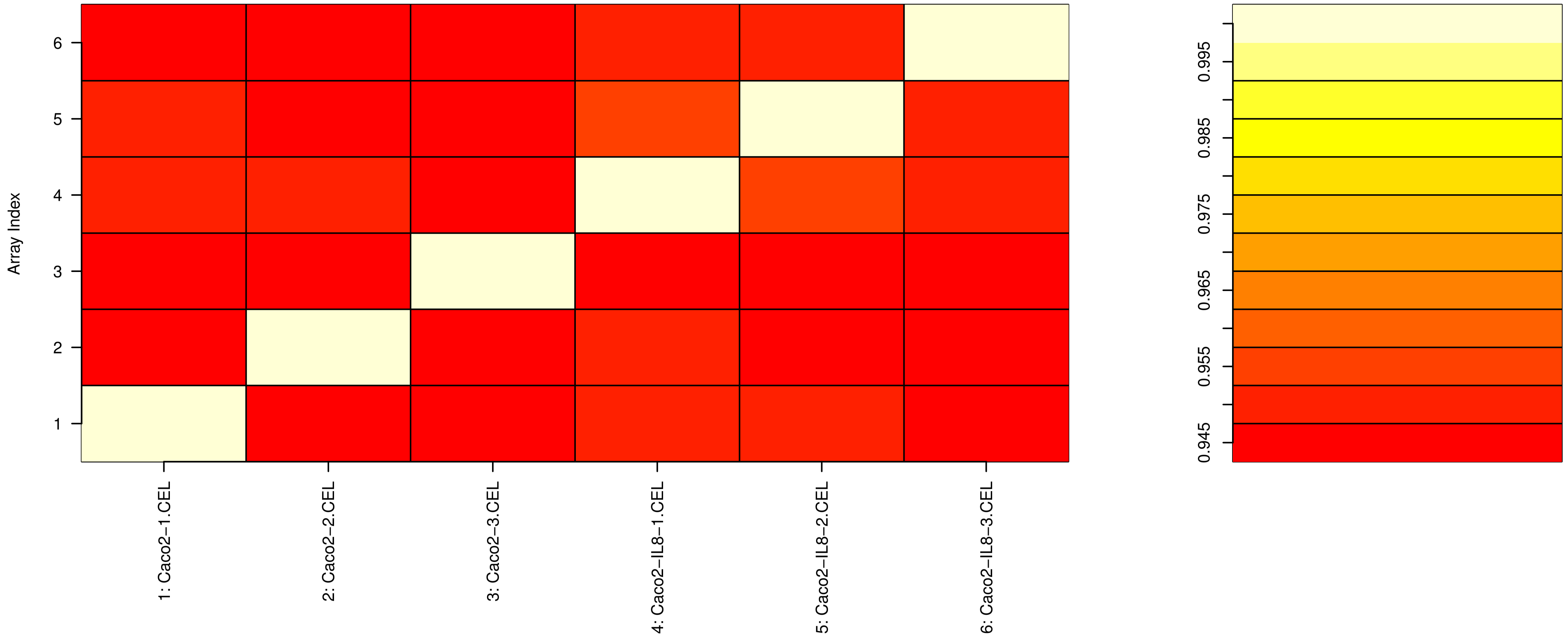

before normalization. Date: Fri Jan 22 15:

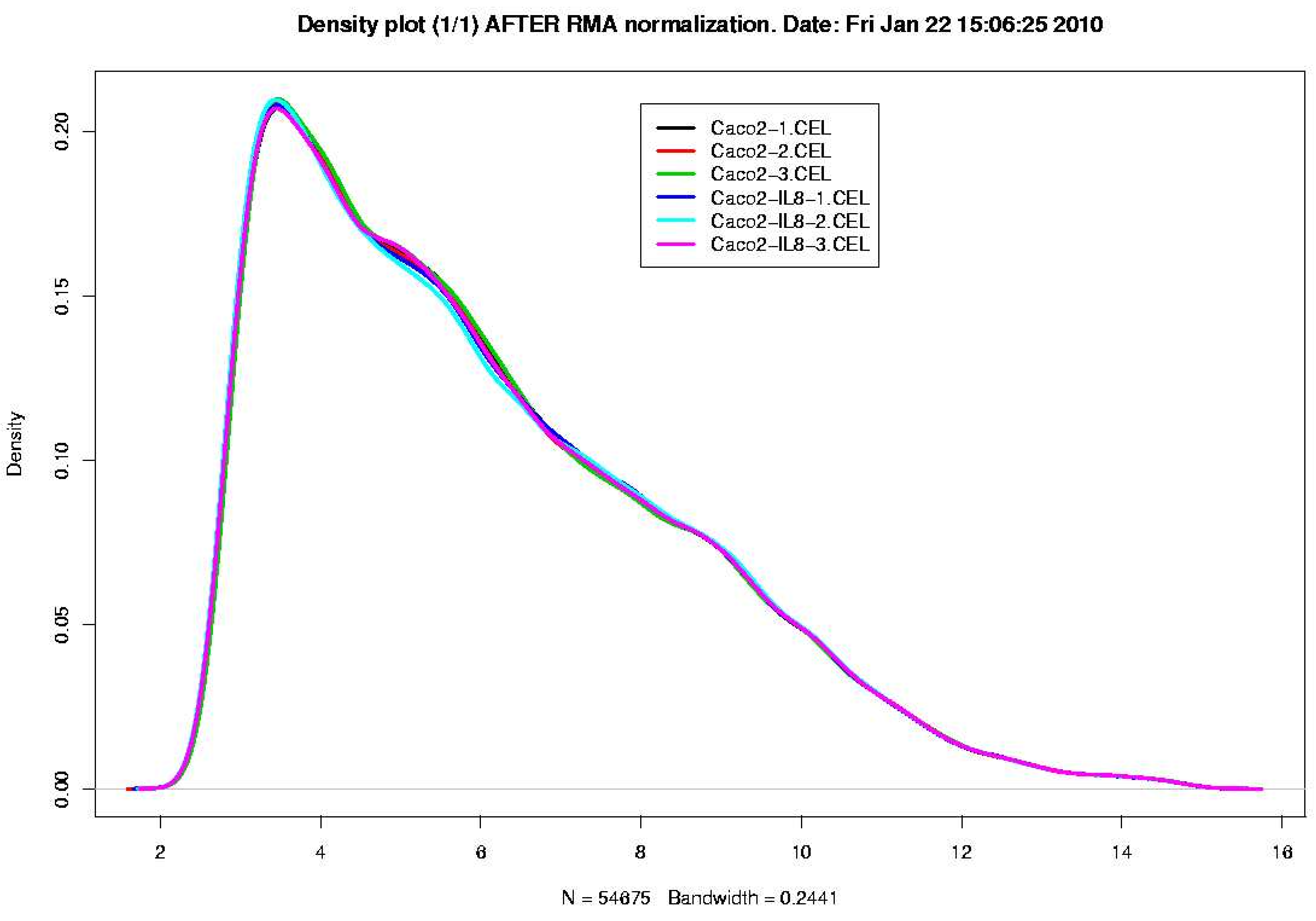
Density plot (1/1) AFTER RMA normalization. Date: Fri Jan 22 15:06:25 2010
Density
0.00
0.05
0.10
0.15
0.20
2
4
6
8
10
12
14
16
N = 54675 Bandwidth = 0.2441
Caco2−1.CEL
Caco2−2.CEL
Caco2−3.CEL
Caco2−IL8−1.CEL
Caco2−IL8−2.CEL
Caco2−IL8−3.CEL

Density plot of ALL arrays AFTER RMA normalization. Date: Fri Jan 22 15:06:27 2010

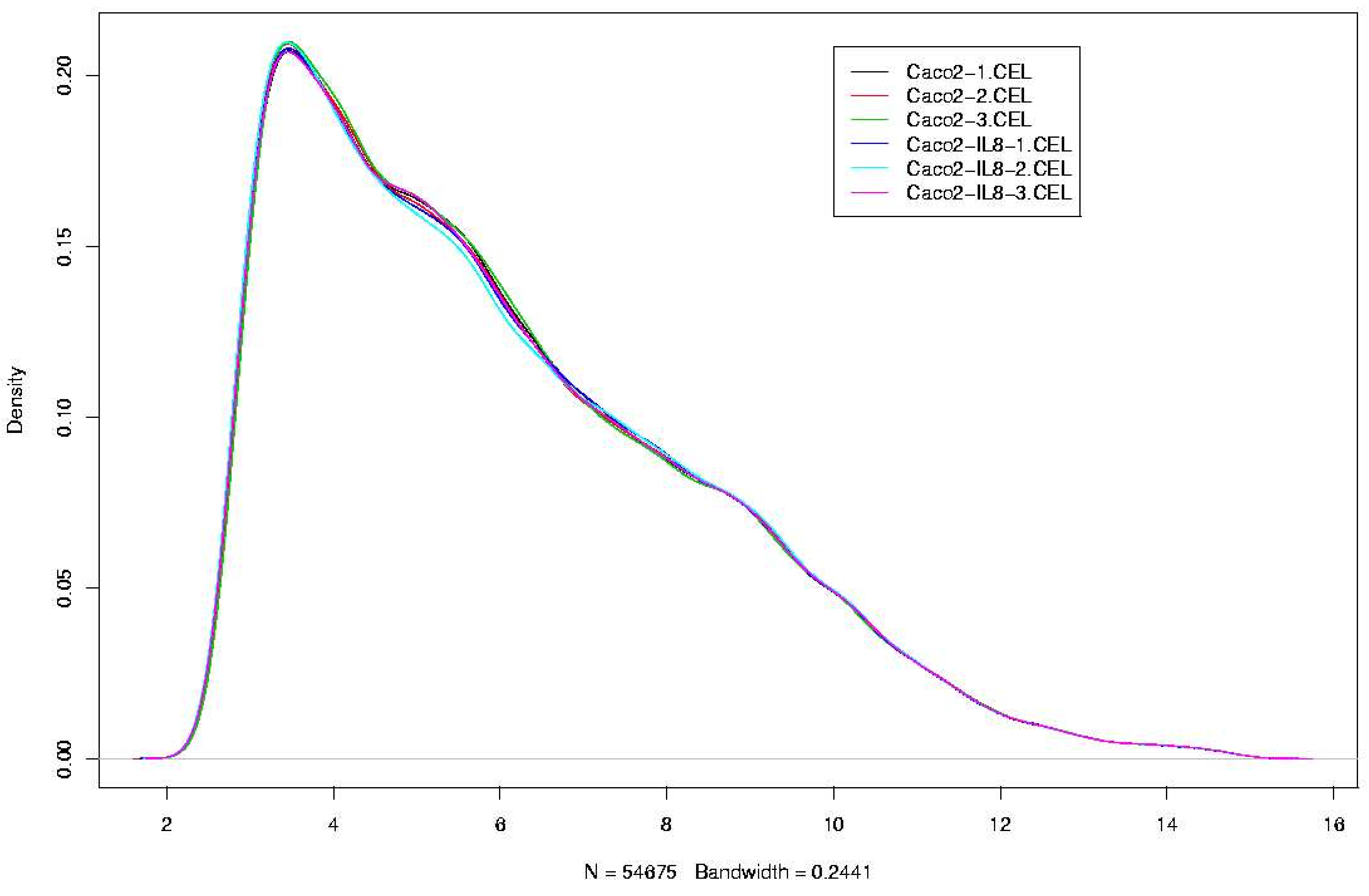

Boxplot AFTER RMA normalization. Date: Fri Jan 22 15:06:30 2010

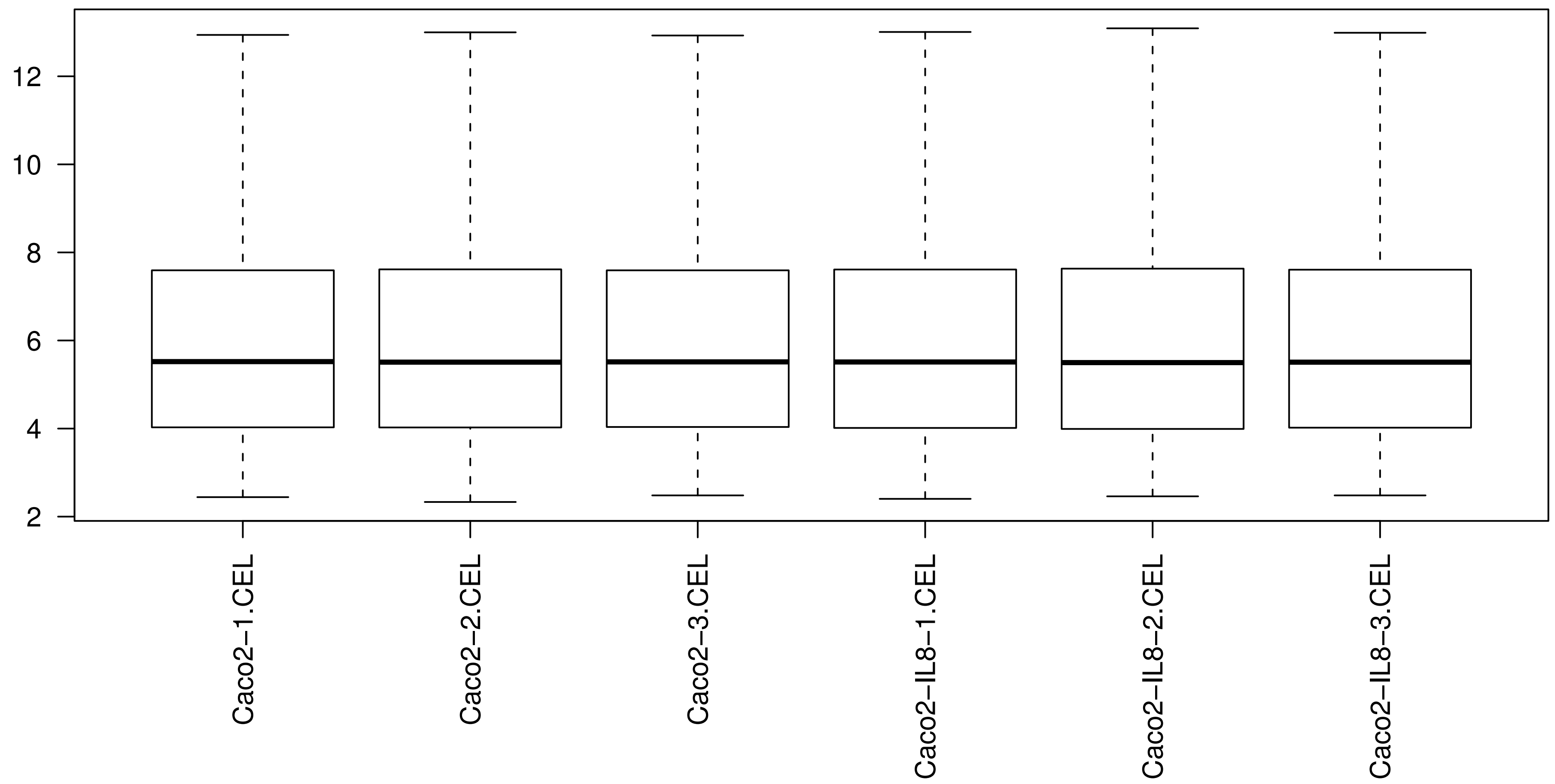

RLE plot (RMA). Date: Fri Jan 22 14:54:04 2010.

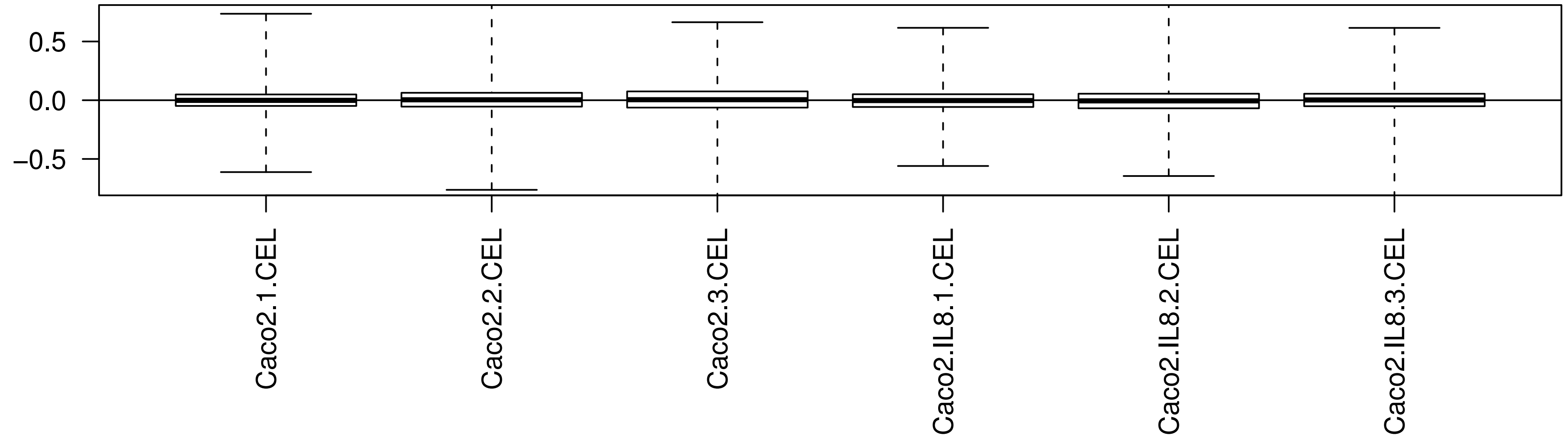

RLE plot (gcRMA(slow)). Date: Fri Jan 22 15:01:20 2010.

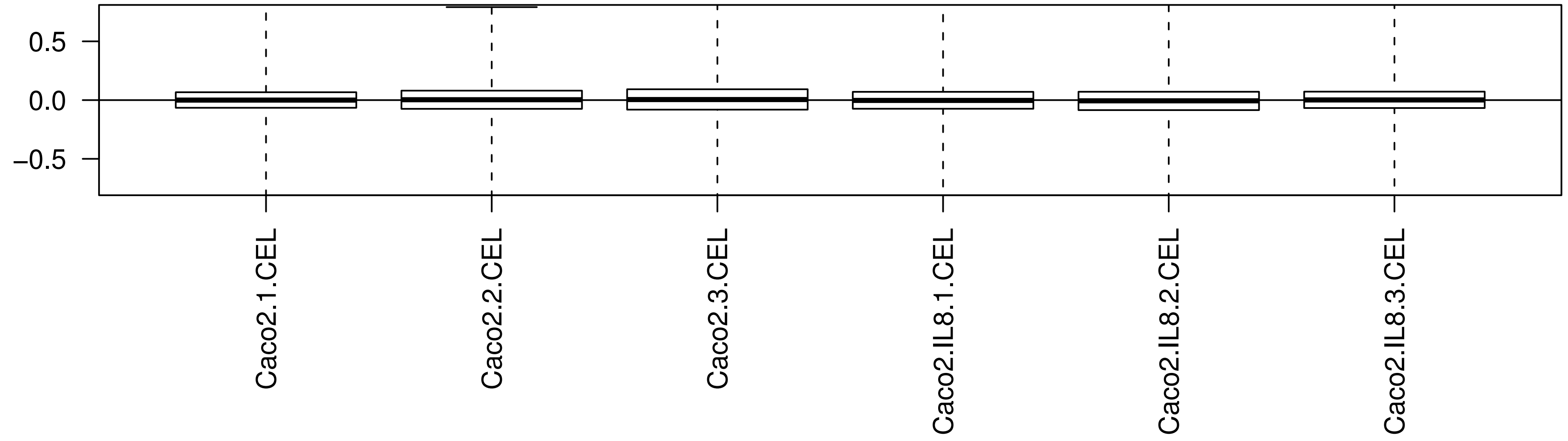

NUSE plot (RMA). Date: Fri Jan 22 14:54:17 2010.

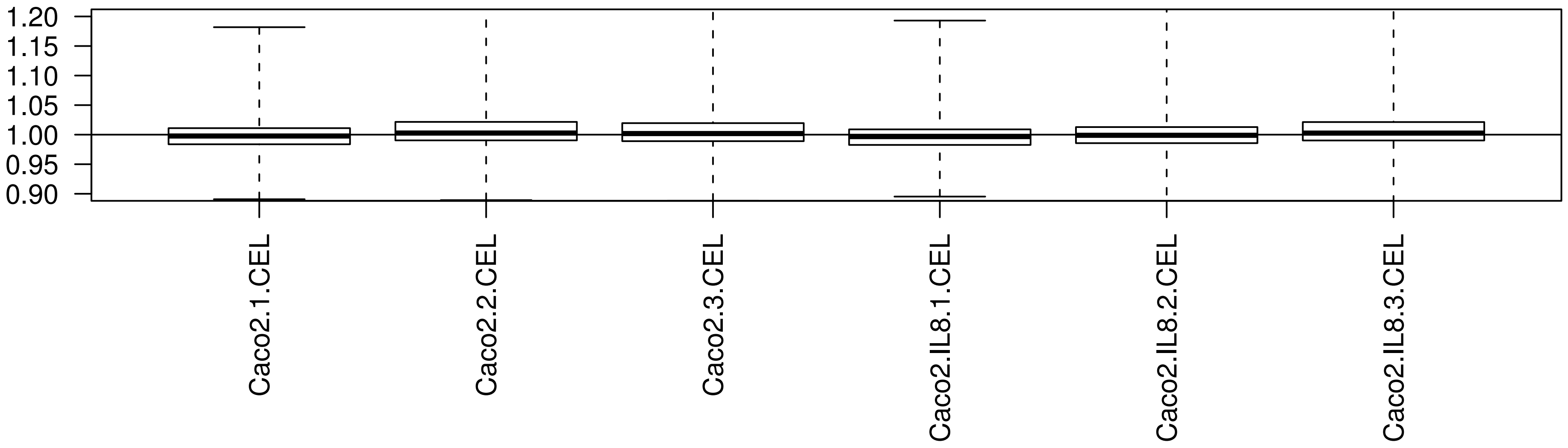

NUSE plot (gcRMA(slow)). Date: Fri Jan 22 15:01:06 2010.

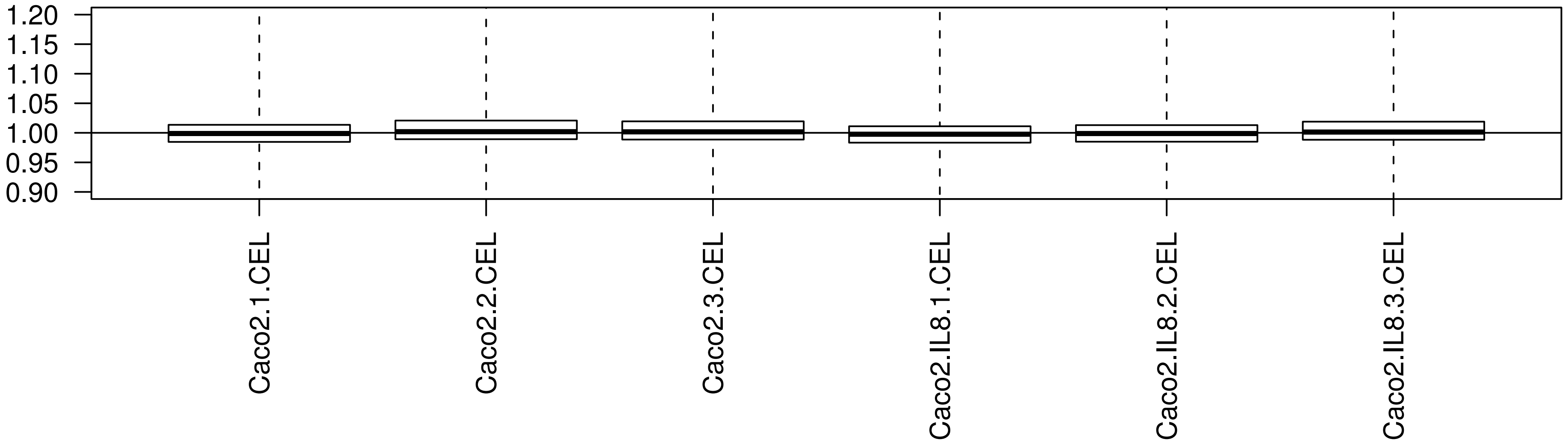

Array–Array Intensity Correlation: Correlation plot AFTER RMA normalisation (Fri Jan 22 15:07:01 2010).

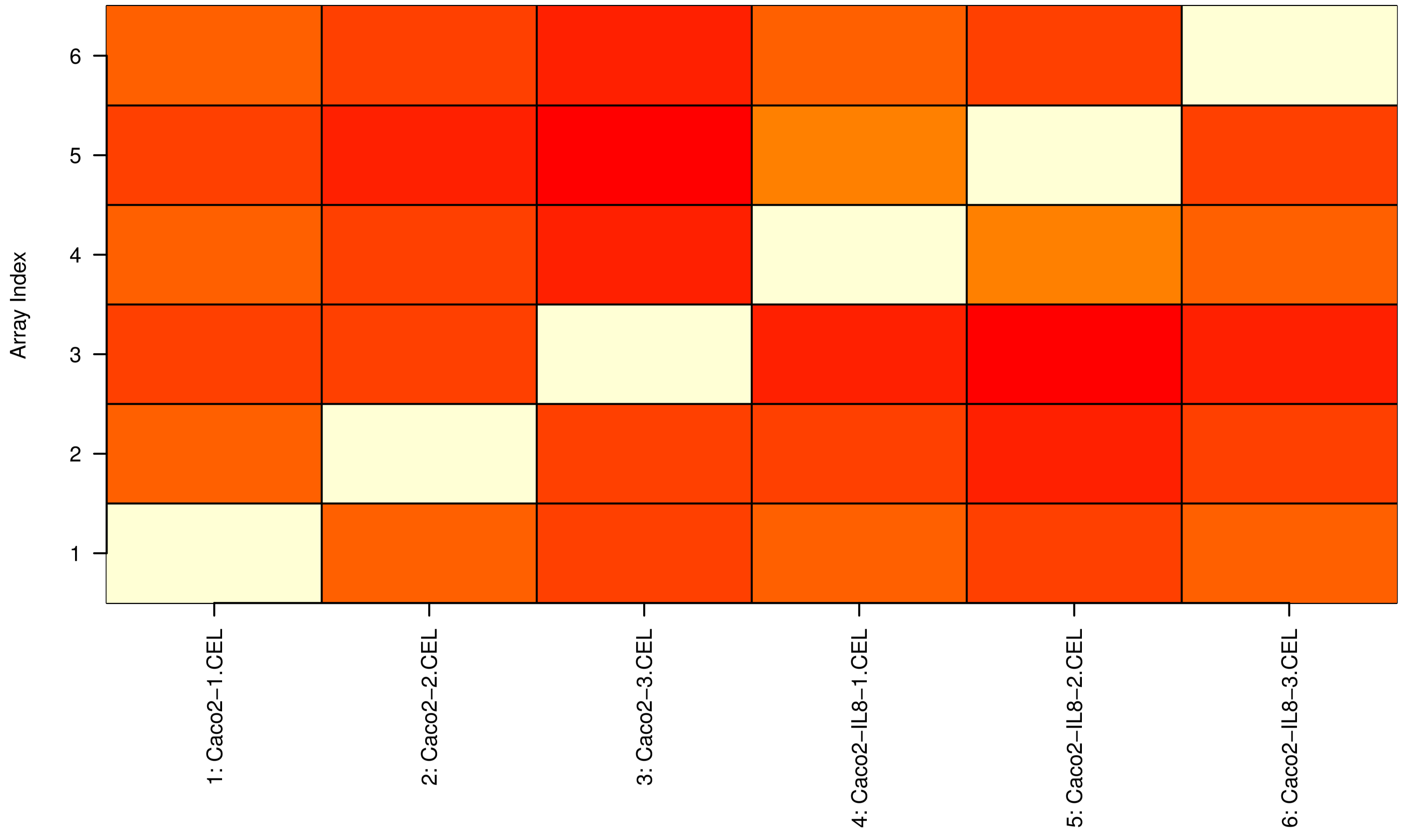

ıfter RMA normalization. Date: Fri Jan 22 15

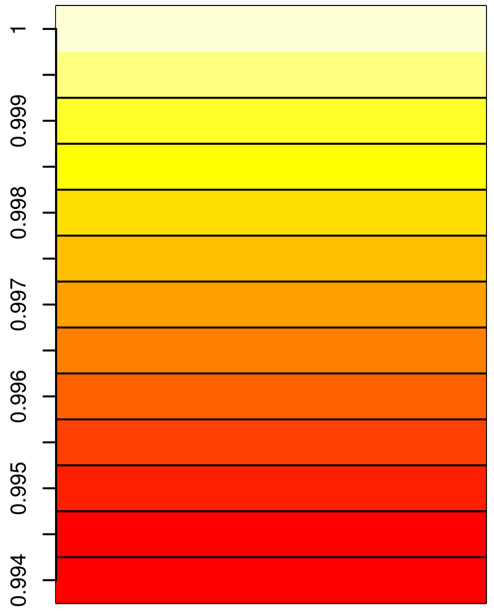

Dendrogram using Ward clustering on the Pearson distance measure (RMA). Date: Fri Jan 22 15:07:02 2010.

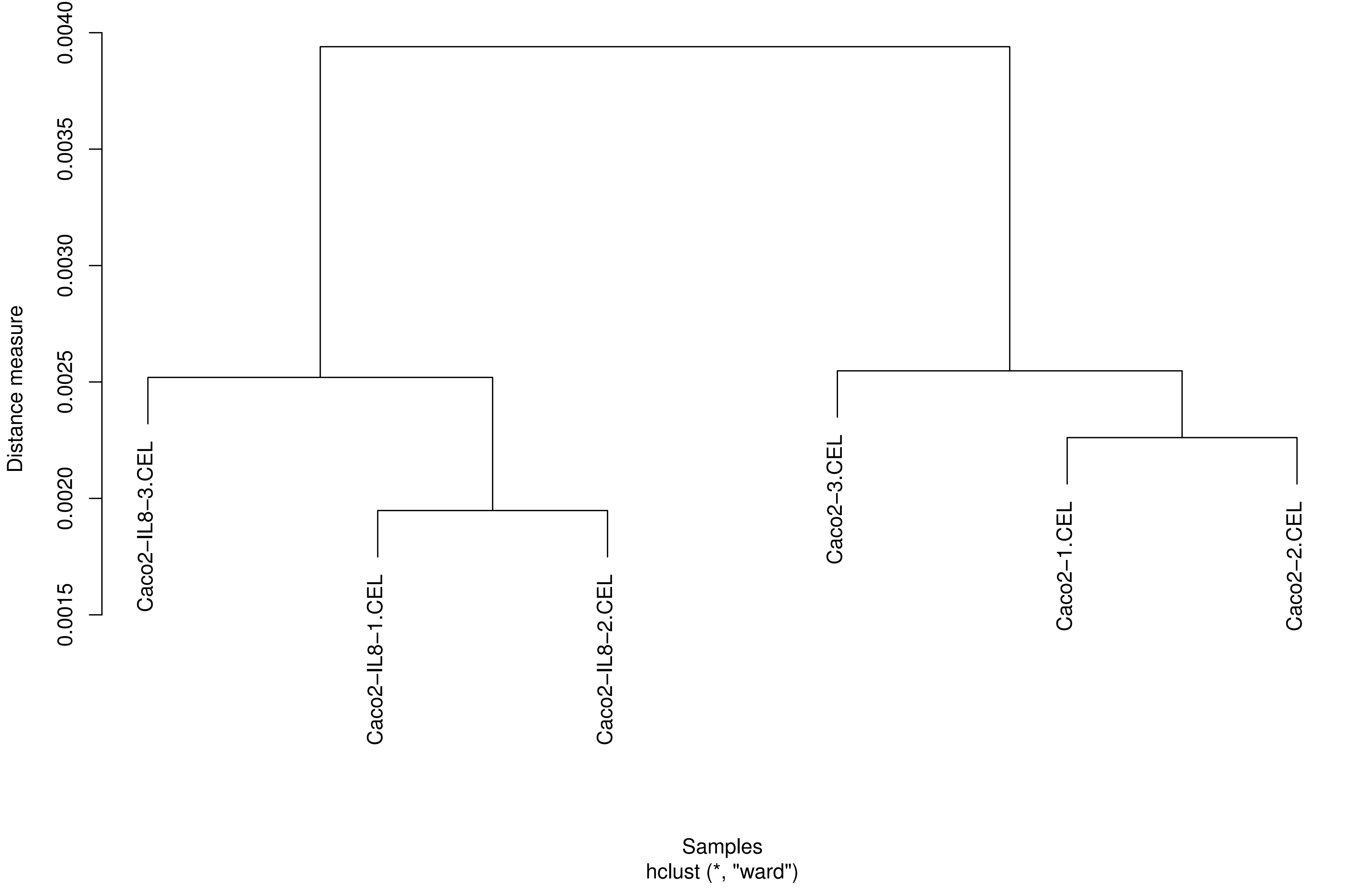

Supplement: Additional file 1 — Supplementary Quality Control Report: Outcome of quality control analyses performed using Bioconductor ( http://www.bioconductor.org ). [file 1756-0500-6-431-S1.pdf]
